# Sacubitril/valsartan and quality of life assessed using the EuroQol Five-dimension Three-level questionnaire level sum score (EQ-5D-3L-LSS) in patients with HFrEF and HFmrEF/HFpEF

**DOI:** 10.1093/ehjcvp/pvaf064

**Published:** 2025-08-21

**Authors:** Mingming Yang, Alasdair D Henderson, Inder S Anand, Akshay S Desai, Carolyn S P Lam, Aldo P Maggioni, Felipe A Martinez, Jean L Rouleau, Karl Swedberg, Muthiah Vaduganathan, Dirk J van Veldhuisen, Faiez Zannad, Michael R Zile, Milton Packer, Adel Rizkala, Eldrin F Lewis, Pardeep S Jhund, Scott D Solomon, John J V McMurray

**Affiliations:** British Heart Foundation Cardiovascular Research Centre, University of Glasgow, 126 University Place, Glasgow G12 8TA, UK; Department of Cardiology, Zhongda Hospital, School of Medicine, Southeast University, Nanjing 210009, China; British Heart Foundation Cardiovascular Research Centre, University of Glasgow, 126 University Place, Glasgow G12 8TA, UK; Department of Cardiovascular Medicine, University of Minnesota, Minneapolis, MN 55455, USA; Cardiovascular Division, Brigham and Women’s Hospital, Harvard Medical School, Boston, MA 02115, USA; National Heart Centre Singapore & Duke-National University of Singapore 169609, Singapore; ANMCO Research Center, Heart Care Foundation, Florence 50121, Italy; Universidad Nacional de Córdoba, Córdoba X5000, Argentina; Institut de Cardiologie de Montréal, Université de Montréal, Montréal H1T 1C8, Canada; Department of Emergency and Cardiovascular Medicine, Sahlgrenska Academy, University of Gothenburg, Gothenburg 40530, Sweden; Cardiovascular Division, Brigham and Women’s Hospital, Harvard Medical School, Boston, MA 02115, USA; Department of Cardiology, Thorax Center, University Medical Center, Postbus 30.001, 9700 RB Groningen, the Netherlands; Inserm Clinical Investigation Centre, CHU, Université de Lorraine, Nancy 54500, France; RHJ Department of Veterans Affairs Medical Center, Medical University of South Carolina, Charleston, SC 29401, USA; Baylor Heart and Vascular Institute, Baylor University Medical Center, Dallas, TX 75226, USA; Novartis Pharmaceuticals Corporation, East Hanover, NJ 07936, USA; Division of Cardiovascular Medicine, Stanford University School of Medicine, Stanford University, Palo Alto, CA 94305, USA; British Heart Foundation Cardiovascular Research Centre, University of Glasgow, 126 University Place, Glasgow G12 8TA, UK; Cardiovascular Division, Brigham and Women’s Hospital, Harvard Medical School, Boston, MA 02115, USA; British Heart Foundation Cardiovascular Research Centre, University of Glasgow, 126 University Place, Glasgow G12 8TA, UK

**Keywords:** Quality of life, EQ-5D-3L, Sacubitril/valsartan, Heart failure, Symptoms

## Abstract

**Aims:**

To investigate the EQ-5D-3L Level Sum Score (LSS) in patients with heart failure (HF) and reduced (HFrEF) and mildly reduced or preserved ejection fraction (HFmrEF/HFpEF) and the effect of sacubitril/valsartan on this score using patient-level data from the PARADIGM-HF and PARAGON-HF trials.

**Methods and results:**

The LSS was calculated by summing the three levels (1–3) for each of the five domains (minimum sum score = 5; maximum sum score = 15). Patient characteristics and outcomes were compared across LSS tertiles (T1–T3) at baseline. Cox models were used to evaluate the primary endpoint [first HF hospitalization or cardiovascular death (CVD)] according to tertiles of LSS. Changes in LSS severity at 8 months were analysed using ordinal logistic regression models to estimate the effect of sacubitril/valsartan vs. enalapril or valsartan. Of 13 195 patients, 12 974 had a baseline LSS. Compared to lower LSS, patients with higher (worse) scores were older, more often women and White, and had more comorbidities and more severe HF. At 8 months, patients assigned to sacubitril/valsartan experienced more improvement and less worsening of LSS vs. the comparator: OR:1.16 (95%CI: 1.08–1.24). Sacubitril/valsartan also reduced the risk of the primary outcome across LSS tertiles: T1: HR: 0.87 (95%CI: 0.75–1.00); T2: 0.80 (95%CI: 0.71–0.90); T3: 0.87 (95%CI: 0.77–0.97); *P*_interaction_ = 0.59. Higher LSS was independently associated with a greater risk of the primary endpoint, and the achieved LSS at 8 months may be more strongly associated with subsequent outcomes.

**Conclusion:**

Sacubitril/valsartan significantly reduced the risk of HF events and improved health status across the LSS spectrum in HFrEF and HFmrEF/HFpEF.

**Clinical Trial Registration:**

https://www.clinicaltrials.gov. Unique identifiers: NCT01920711 (PARAGON-HF), NCT01035255 (PARADIGM-HF).

## Graphical Abstract

**Graphical Abstract:**
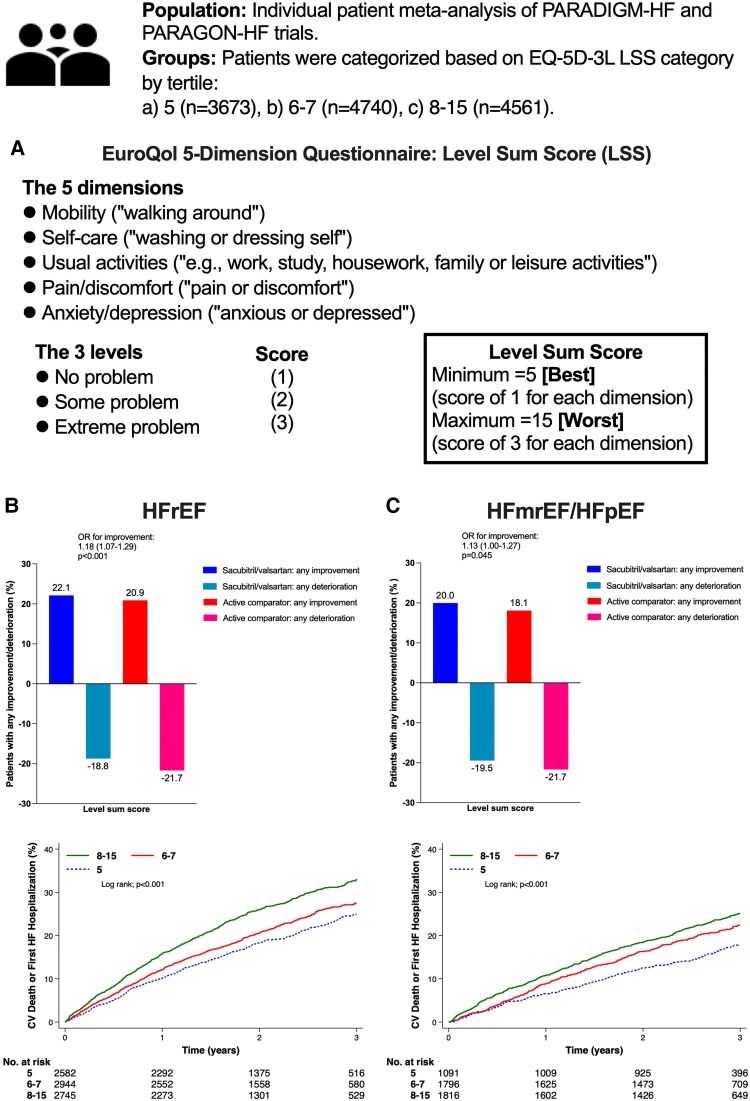
Sacubitril/valsartan and health status measured using the EuroQol Five-Dimension Three-Level (EQ-5D-3L) questionnaire Level Sum Score (LSS) in patients with HFrEF and HFpEF. All the EQ-5D and severity levels contribute equally when calculating the LSS (A). Irrespective of HF phenotype, sacubitril/valsartan compared to a renin–angiotensin system (RAS) comparator (enalapril or valsartan) improved patient health status between baseline and 8 months as assessed with LSS (B-HFrEF and C-HFmrEF/HFpEF). Worse baseline EQ-5D-3L-LSS was associated with a higher risk of subsequent clinical outcomes. HF, heart failure; HFpEF, heart failure with preserved ejection fraction; OR, odds ratio; RAS, renin–angiotensin system.

## Introduction

Heart failure (HF) is a progressive condition associated with significant morbidity, mortality, and poor health-related quality of life (HRQL).^1^ While clinical outcomes such as hospitalization and mortality remain key endpoints in HF trials, HRQL is increasingly recognized as an important measure of therapeutic efficacy.^2-4^ The EuroQol Five-Dimension Three-Level (EQ-5D-3L) questionnaire is a standardized, validated, and widely used instrument for assessing generic HRQL across various conditions, although there are few data in HF.^1,5-8^ In practice, EQ-5D is valued for its brevity and utility indexing (for cost-effectiveness).^9-11^ The Level Sum Score (EQ-5D-3L-LSS), derived from the EQ-5D-3L, provides a composite measure of health status by summing patient responses on three severity levels (1=‘no problem’, 2=‘some problem’, 3=‘extreme problem’) across five dimensions: mobility, self-care, usual activities, pain/discomfort, and anxiety/depression.^12^

Studies have demonstrated that higher EQ-5D scores (worse HRQL) are associated with a greater risk of adverse clinical outcomes in patients with HF, irrespective of left ventricular ejection fraction (LVEF).^13-17^ However, there are few data on the effects of therapeutic interventions for HF on EQ-5D. Early evidence from the CARE-HF trial demonstrated that cardiac resynchronization therapy (CRT) significantly improved quality of life, including EQ-5D scores, and that these benefits were sustained over long-term follow-up.^18,19^ Similarly, in the HF-ACTION trial, aerobic exercise training led to a modest improvement in EQ-5D visual analogue scale (VAS) score compared with usual care at 3 months.^20^ More recently, in the DAPA-HF and DELIVER trials, dapagliflozin improved EQ-5D scores and multiple HRQL dimensions, including mobility, self-care, usual activities, and possibly anxiety/depression.^16,17^ The EQ-5D was also employed in two trials using sacubitril/valsartan, an angiotensin receptor-neprilysin inhibitor (ARNI), to evaluate patient-reported health status. The first, the PARADIGM-HF trial (Prospective Comparison of ARNI With ACEI to Determine Impact on Global Mortality and Morbidity in Heart Failure), demonstrated superiority over an angiotensin-converting enzyme inhibitor in patients with HF and reduced LVEF (HFrEF).^21^ In patients with HF and mildly reduced or preserved LVEF (HFmrEF/HFpEF), the PARAGON-HF trial (Prospective Comparison of ARNI With ARB Global Outcomes in HF With Preserved Ejection Fraction) showed that sacubitril/valsartan led to a non-significant reduction in the composite endpoint of total HF hospitalizations and cardiovascular mortality when compared with an angiotensin II receptor blocker [rate ratio, 0.87; 95% confidence interval (CI), 0.75–1.01; *P* = 0.06].^22,23^

In the present study, we examined the effects of sacubitril/valsartan on EQ-5D-3L-LSS and its dimensions over time in patients with HFrEF and HFmrEF/HFpEF, using data from PARADIGM-HF and PARAGON-HF trials. We also evaluated the relationship between baseline EQ-5D-3L scores and patient characteristics.

## Methods

### Trials and patients

In this *post hoc* analysis, we conducted a pooled analysis of patient-level data from PARADIGM-HF (NCT01035255) and PARAGON-HF (NCT01920711). The study designs, baseline characteristics, and primary outcomes of these trials have been published.^21,22^ In brief, both PARADIGM-HF and PARAGON-HF were multinational, multicentre, phase 3, randomized, double-blind clinical trials that assessed the efficacy and safety of sacubitril/valsartan compared to a renin–angiotensin system (RAS) comparator (enalapril or valsartan, respectively) in patients with HF and LVEF ≤ 40% (HFrEF, PARADIGM-HF) or LVEF ≥ 45% (HFmrEF/HFpEF, PARAGON-HF). In PARADIGM-HF, sacubitril/valsartan was compared to enalapril, a well-established and effective therapy for HFrEF, whereas in PARAGON-HF, it was compared to valsartan, which is not an established treatment for HFpEF and whose efficacy in HFmrEF remains uncertain. The key inclusion and exclusion criteria are summarized in the Supplementary Material, and a complete list of inclusion and exclusion criteria is available in the primary results publications.^21,22^ All patients provided written informed consent, and both study protocols were approved by Ethics Committees at each participating site.

### European quality-of-life questionnaire five dimensions three levels: EuroQoL (EQ-5D-3L)

The EQ-5D-3L questionnaire consists of two sections: a descriptive section and a visual analogue scale (EQ-VAS) (*Graphical abstract*). The questionnaire was self-administered and the EQ-5D-3L-LSS was derived by summing the responses across all five dimensions, providing a composite measure of HRQL with possible scores ranging from 5 (best possible health status) to 15 (worst possible status).^12^

### Clinical outcomes

The primary endpoint in PARADIGM-HF was the composite of CV death or first HF hospitalization, and in PARAGON-HF, a composite of total (first and recurrent) HF hospitalizations and CV death causes.^21,22^ In the present analyses, we examined both outcomes and the time to first HF hospitalization, and death (due to CV, non-CV, and all causes).

### Statistical analysis

Baseline characteristics were summarized using medians with interquartile ranges (Q1–Q3) for continuous variable and frequencies with percentages for categorical variables according to the EQ-5D-3L-LSS tertiles (T1–T3). Group comparisons were conducted with the Jonckheere-Terpstra test for continuous variables and the Cochran–Armitage test for a binary variable to assess trends across different EQ-5D-3L-LSS groups.

The cumulative incidence of time-to-first-event outcome according to each LSS tertile and severity levels of individual dimensions was estimated using the Kaplan–Meier method. The association between EQ-5D-3L-LSS and each outcome was evaluated using Cox proportional hazards regression models with patients in EQ-5D-3L-LSS T1 (i.e. LSS = 5) as the reference group, stratified according to geographic region and study. Negative binomial regression analysis, adjusted for geographic region and trial with an offset for follow-up time, was performed to compare the composite of total (first and repeat) HF hospitalizations and CV death, across EQ-5D categories. The above analyses also adjusted for: age, sex, heart rate, systolic blood pressure (SBP), body mass index (BMI), New York Heart Association (NYHA) functional class, LVEF, estimated glomerular filtration rate (eGFR), N-terminal pro B-type natriuretic peptide (NT-proBNP, log-transformed), atrial fibrillation, history of myocardial infarction (MI), diabetes, and stroke.

The effect of sacubitril/valsartan compared to a RAS inhibitor was calculated as a rate ratio (RR) and 95% confidence interval (95%CI) derived from negative binomial regression models for total (first and recurrent) events or as a hazard ratio (HR) and 95%CI from Cox proportional hazards models for time-to-first events.

To examine the longitudinal changes in EQ-5D scores, individual patient trajectories were visualized using Sankey diagrams, which captured shifts in severity categories from baseline to the 8-month follow-up assessment.

The treatment effects of sacubitril/valsartan on EQ-5D scores were assessed at baseline and 8-month follow-up, with no imputation for missing data. Changes in EQ-5D severity categories at 8 months were classified as ‘improved’, ‘no change’, or ‘worsened’. An ordinal logistic regression model was used to evaluate the effect of sacubitril/valsartan on changes in EQ-5D scores, with results reported as overall odds ratios (ORs) representing the likelihood of experiencing improvement vs. deterioration in EQ-5D scores. The same statistical approach was applied to analyse EQ-5D-3L-LSS categories. A subgroup analysis was performed to assess the effect of sacubitril/valsartan on EQ-5D-3L-LSS by sex (men and women) given the previously observed effect modification by sex in the PARAGON-HF trial.^22^

All statistical analyses were conducted using Stata/SE version 18.0 (Stata Corp, College Station, TX). A two-tailed *P*-value <0.05 was considered statistically significant for all tests.

## Results

Of the 13 195 patients randomized in PARADIGM-HF and PARAGON-HF, 12 974 completed the EQ-5D-3L questionnaire at baseline, and 11 915 (90.3%) at the 8-month follow-up visit (see Supplementary material online, *Table S1*). The distribution of responses across the five EQ-5D-3L dimensions for the entire cohort is detailed in Supplementary material online, *Table S1* and Supplementary material online, *Figure S1*, while the distributions for each trial are shown in Supplementary material online, *Table S2* (PARADIGM-HF) and 3 (PARAGON-HF), respectively. The categorization of patients based on individual EQ-5D-3L-LSS is illustrated in Supplementary material online, *Figure S2*, while stratifications by tertiles are presented in Supplementary material online, *Table S1* (overall cohort), *Table 1* (HFrEF), *Table 2* (HFmrEF/HFpEF), and the *Graphical abstract*, with additional breakdowns by HF phenotype in Supplementary material online, *Tables S2* and *S3*.

**Table 1:** Baseline characteristics according to baseline EuroQol Five-dimension Three-level (EQ-5D-3L) Questionnaire Level Sum Score (LSS) category divided by tertile in HFrEF

	Tertile 1: 5	Tertile 2: 6–7	Tertile 3: 8–15	*P-*Value for trend
*N* (%)	2582 (31.2)	2944 (35.6)	2745 (33.2)	
Age, yr	63 (56–71)	64 (57–73)	65 (58–73)	<0.001
**Women**	**387 (15.0)**	**587 (19.9)**	**817 (29.8)**	**<0**.**001**
Race				<0.001
White	1506 (58.3)	1882 (63.9)	2098 (76.4)	
Black	141 (5.5)	168 (5.7)	117 (4.3)	
Asian	617 (23.9)	535 (18.2)	295 (10.7)	
Others	318 (12.3)	359 (12.2)	235 (8.6)	
SBP, mmHg	120 (110–130)	120 (110–130)	121 (110–131)	<0.001
BMI, kg/m^2^	27.0 (24.2–30.4)	27.2 (24.4–30.8)	28.4 (25.0–32.5)	<0.001
Atrial fibrillation (history)	825 (32.0)	1028 (34.9)	1214 (44.2)	<0.001
MI	1014 (39.3)	1257 (42.7)	1307 (47.6)	<0.001
Stroke	159 (6.2)	244 (8.3)	309 (11.3)	<0.001
Diabetes mellitus	807 (31.3)	1011 (34.3)	1037 (37.8)	<0.001
Time since HF diagnosis				<0.001
≤1yr	889 (34.4)	902 (30.6)	677 (24.7)	
>1–5yrs	971 (37.6)	1095 (37.2)	1125 (41.0)	
>5yrs	722 (28.0)	947 (32.2)	943 (34.4)	
Previous hospitalization for HF	1558 (60.3)	1842 (62.6)	1796 (65.4)	<0.001
**NYHA III/IV**	**280 (10.8)**	**607 (20.7)**	**1159 (42.3)**	**<0**.**001**
**KCCQ clinical summary score**	**94 (85–98)**	**82 (72–91)**	**60 (47–73)**	**<0**.**001**
NT-proBNP, pg/mL	1473 (862–2876)	1602 (879–3301)	1760 (931–3572)	<0.001
Atrial fibrillation/flutter^b^	1810 (1113–3101)	2067 (1174–3908)	2088 (1209–4215)	0.002
No atrial fibrillation/flutter^b^	1388 (806–2816)	1478 (828–3043)	1577 (826–3160)	0.004
LVEF, %	30 (25–34)	30 (25–34)	31 (26–35)	<0.001
Creatinine, μmol/L	95 (81–112)	97 (82–116)	94 (80–113)	0.44
eGFR, mL/min/1.73m^2^	68 (56–81)	65 (53–79)	65 (52–78)	<0.001
Diuretics	1974 (76.5)	2361 (80.2)	2297 (83.7)	<0.001
Beta-blocker	2416 (93.6)	2742 (93.1)	2542 (92.6)	0.16
MRA	1423 (55.1)	1601 (54.4)	1580 (57.6)	0.07
Pacemaker	292 (11.3)	406 (13.8)	372 (13.6)	0.02
ICD^a^	377 (14.6)	474 (16.1)	374 (13.6)	0.30

Data are presented as median (25–75% quartile) for continuous measures, and *n* (%) for categorical measures. Those with ‘clinically meaningful’ differences (e.g., sex, KCCQ, NYHA Class) are shown in bold text.

BMI, body mass index; CRT-D, cardiac resynchronization therapy with defibrillator; eGFR, estimated glomerular filtration rate; HF, heart failure; HFrEF, heart failure with reduced ejection fraction; ICD, implantable cardioverter defibrillator; KCCQ, Kansas City Cardiomyopathy Questionnaire; LVEF, left ventricular ejection fraction; MI, myocardial infarction; MRA, mineralocorticoid receptor antagonist; NT-proBNP, N-terminal pro B-type natriuretic peptide; NYHA, New York Heart Association; SBP, systolic blood pressure; yr, year.

^a^Including CRT-D.

^b^Based on electrocardiogram.

**Table 2:** Baseline characteristics according to baseline EuroQol Five-Dimension Three-level (EQ-5D-3L) Questionnaire Level Sum Score (LSS) category divided by tertile in HFmrEF/HFpEF

	Tertile 1: 5	Tertile 2: 6–7	Tertile 3: 8–15	*P-*Value for trend
*N* (%)	1091 (23.2)	1796 (38.2)	1816 (38.6)	
Age, yr	72 (66–78)	74 (67–79)	74 (68–79)	<0.001
**Women**	**423 (38.8)**	**891 (49.6)**	**1110 (61.1)**	**<0**.**001**
Race				<0.001
White	776 (71.1)	1477 (82.2)	1573 (86.6)	
Black	23 (2.1)	37 (2.1)	42 (2.3)	
Asian	239 (21.9)	207 (11.5)	150 (8.3)	
Others	53 (4.9)	75 (4.2)	51 (2.8)	
SBP, mmHg	130 (120–140)	130 (120–140)	130 (120–140)	0.40
BMI, kg/m^2^	28.6 (25.4–32.0)	29.8 (26.5–33.4)	30.9 (27.3–35.1)	<0.001
Atrial fibrillation (history)	581 (53.3)	942 (52.5)	992 (54.6)	0.39
MI	226 (20.7)	422 (23.5)	409 (22.5)	0.36
Stroke	90 (8.3)	170 (9.5)	241 (13.3)	<0.001
Diabetes mellitus	413 (37.9)	759 (42.3)	838 (46.1)	<0.001
Time since HF diagnosis				<0.001
≤1yr	508 (46.6)	728 (40.6)	693 (38.3)	
>1–5yrs	347 (31.9)	636 (35.5)	663 (36.7)	
>5yrs	234 (21.5)	430 (24.0)	453 (25.0)	
Previous hospitalization for HF	503 (46.1)	854 (47.6)	884 (48.7)	0.18
**NYHA III/IV**	**92 (8.4)**	**297 (16.5)**	**550 (30.3)**	**<0**.**001**
**KCCQ clinical summary score**	**91 (81–96)**	**78 (67–88)**	**60 (47–72)**	**<0**.**001**
NT-proBNP, pg/mL	891 (468–1542)	939 (466–1657)	878 (464–1612)	0.73
Atrial fibrillation/flutter^b^	1539 (1162–2246)	1559 (1175–2291)	1672 (1166–2325)	0.26
No atrial fibrillation/flutter^b^	603 (377–992)	603 (387–1136)	596 (375–1025)	0.69
LVEF, %	56 (50–62)	57 (50–62)	57 (51–63)	0.006
Creatinine, μmol/L	94 (78–112)	93 (77–112)	91 (76–111)	0.037
eGFR, mL/min/1.73m^2^	62 (50–77)	61 (49–74)	59 (47–73)	<0.001
Diuretics	1031 (94.5)	1707 (95.0)	1759 (96.9)	0.001
Beta-blocker	864 (79.2)	1443 (80.3)	1443 (79.5)	0.97
MRA	309 (28.3)	439 (24.4)	473 (26.0)	0.30
Pacemaker	79 (7.2)	168 (9.4)	203 (11.2)	<0.001
ICD^a^	4 (0.4)	8 (0.4)	6 (0.3)	0.81

Data are presented as median (25–75% quartile) for continuous measures, and n (%) for categorical measures. Those with ‘clinically meaningful’ differences (e.g., sex, KCCQ, NYHA Class) are in shown in bold text.

BMI, body mass index; CRT-D, cardiac resynchronization therapy with defibrillator; eGFR, estimated glomerular filtration rate; HF, heart failure; HFmrEF, heart failure with mildly reduced ejection fraction; HFpEF, heart failure with preserved ejection fraction; ICD, implantable cardioverter defibrillator; KCCQ, Kansas City Cardiomyopathy Questionnaire; LVEF, left ventricular ejection fraction; MI, myocardial infarction; MRA, mineralocorticoid receptor antagonist; NT-proBNP, N-terminal pro B-type natriuretic peptide; NYHA, New York Heart Association; SBP, systolic blood pressure; yr, year.

^a^Including CRT-D.

^b^Based on electrocardiogram.

### Patient characteristics according to EQ-5D-3L-LSS

Baseline characteristics according to EQ-5D-3L-LSS are summarized in *Table 1* (HFrEF), *Table 2* (HFmrEF/HFpEF), and Supplementary material online, *Tables S4* and *S5* (overall cohort). Same results for EQ-5D VAS are reported in Supplementary material online, *Table S6*.

#### Demographic and clinical characteristics

Compared to patients in the best LSS tertile (lowest scores, best HRQL), those in worse LSS tertiles (higher scores, worse HRQL) were generally older, and more frequently female and White. They also had a higher heart rate and SBP, and higher proportions of obesity and other comorbidities, including atrial fibrillation, coronary artery disease, stroke, diabetes, lung disease, anaemia, and chronic kidney disease (CKD).

#### Heart failure characteristics and treatments

Compared to patients with worse EQ-5D-3L-LSS, patients in better LSS tertiles were more likely to have longer duration HF, higher LVEF, and more severe HF, as indicated by worse NYHA functional class and lower Kansas City Cardiomyopathy Questionnaire (KCCQ) and EQ-VAS scores. They also had lower eGFR. Interestingly, NT-proBNP levels did not differ meaningfully across tertiles of baseline EQ-5D-3L-LSS, with no significant trend observed (*P* for trend = 0.45).

### EQ-5D-3L individual dimension scores

The distribution of EQ-5D-3L responses across individual dimensions is shown in Supplementary material online, *Tables S1*–S3 and Supplementary material online, *Figure S1*. The proportion of patients reporting no/some/extreme problems was 52.9/46.9/0.2% for mobility (‘walking about’), 83.7/15.8/0.6% for self-care (‘washing or dressing myself’); 57.3/40.3/2.5% for usual activities (‘work, study, housework, family or leisure activities’), 52.9/44.3/2.8% for pain/discomfort, and 67.6/30.5/1.9% for anxiety/depression (see Supplementary material online, *Figure S1*). Compared with patients with HFrEF, those with HFmrEF/HFpEF reported more problems in the mobility and pain/discomfort dimensions. Specifically, 53.6% of patients with HFmrEF/HFpEF reported some problems with mobility compared with 43.1% in HFrEF, and 48.4% of patients with HFmrEF/HFpEF reported some problems with pain/discomfort vs. 41.9% in HFrEF. A greater proportion of patients with HFmrEF/HFpEF also reported extreme problems with pain/discomfort (4.4% vs. 1.9%). In contrast, the proportions reporting issues in self-care, usual activities, and anxiety/depression were generally similar between the two groups (see Supplementary material online, *Table S7*).

The baseline characteristics according to responses to each EQ-5D-3L dimension are presented in Supplementary material online, *Tables S8*–S12. Compared to patients who reported no issues in any dimension (T1, LSS = 5), those experiencing greater impairment were generally older, more frequently female, and exhibited a higher prevalence of comorbidities, along with characteristics suggesting more severe HF.

### Effect of sacubitril/valsartan on EQ-5D-3L individual dimension scores

**Figure 1:**
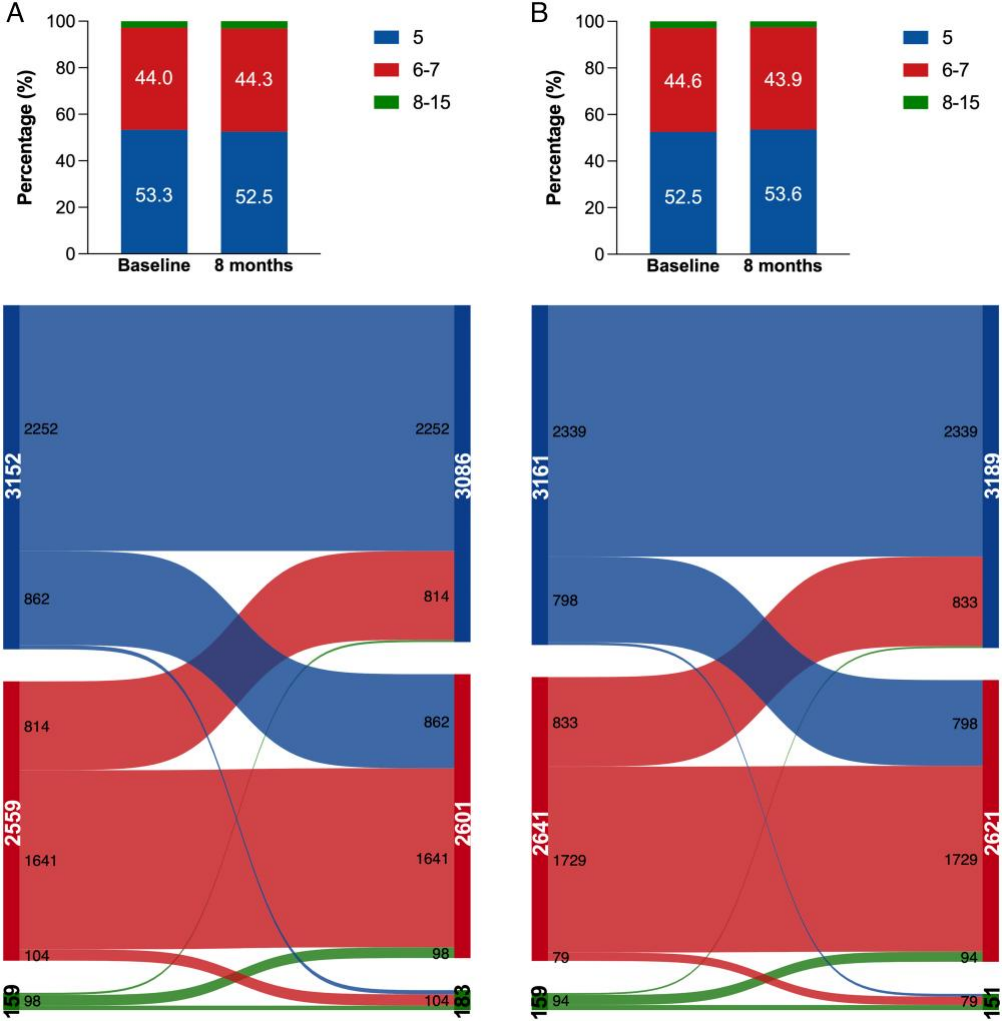
Distribution and changes in answers to the EuroQol Five-dimension Three-level (EQ-5D-3L) ‘pain/discomfort’ question from baseline to 8-month follow-up. (*A*) Distribution and Sankey plot for the active comparator group; (*B*) Distribution and Sankey plot for the Sacubitril/Valsartan group. Only values ≥ 50 in the subgroups were shown in the Sankey plot. Bars represent the number of patients in each EQ-5D-5L category. Colours indicate the distribution of EQ-5D-3L scores at randomization and after 8 months of follow-up. Patients with missing EQ-5D-3L assessments are not displayed.

**Figure 2:**
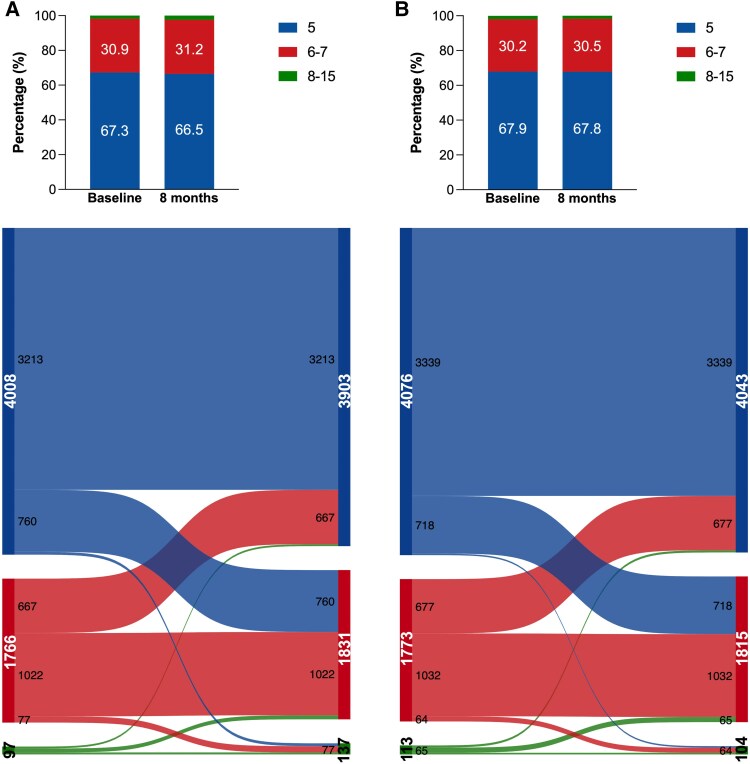
Distribution and changes in answers to the EuroQol Five-dimension Three-level (EQ-5D-3L) ‘anxiety/depression’ question from baseline to 8-month follow-up. (*A*) Distribution and Sankey plot for the active comparator group; (*B*) Distribution and Sankey plot for the Sacubitril/Valsartan group. Only values ≥ 50 in the subgroups were shown in the Sankey plot. Bars represent the number of patients in each EQ-5D-3L category. Colours indicate the distribution of EQ-5D-3L scores at randomization and after 8 months of follow-up. Patients with missing EQ-5D-3L assessments are not displayed.

**Figure 3:**
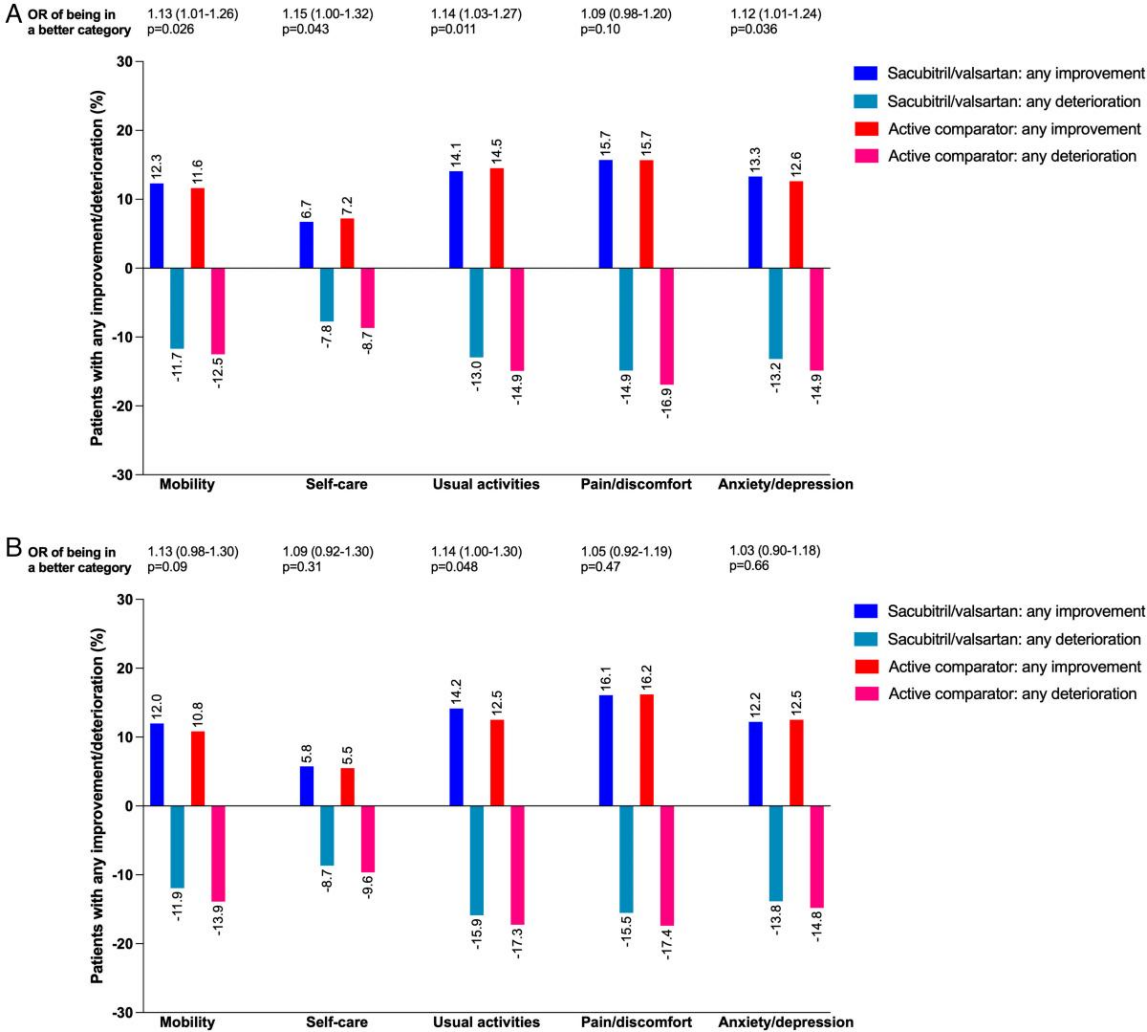
Effect of sacubitril/valsartan vs. active comparator on EuroQol Five-dimension Three-level (EQ-5D-3L) Questionnaire dimension over time (8 months). (*A*) HFrEF, (*B*) HFmrEF/HFpEF.

Changes in EQ-5D-3L dimension scores from baseline to 8 months are shown in *Figures 1* and *2* (pain/discomfort, and anxiety/depression) and Supplementary material online, *Figures S3*–S5 (mobility, self-care, usual activities). Treatment with sacubitril/valsartan was associated with a reduced likelihood of deterioration in all dimensions by 8 months: mobility: 11.8% vs. 13.0%, self-care: 8.1% vs. 9.0%, usual activities: 14.0% vs. 15.8%, pain/discomfort:15.1% vs. 17.1%, and anxiety/depression: 13.4% vs. 14.9% (see Supplementary material online, *Figure S6*, *Figure 3*). Patients treated with sacubitril/valsartan demonstrated a greater improvement in mobility scores compared to placebo (12.2% vs. 11.3%). Individuals randomized to sacubitril/valsartan were more likely to experience improvement rather than deterioration across multiple EQ-5D, including mobility (OR: 1.13, 95%CI: 1.04–1.23; *P* = 0.005), self-care (1.12, 1.01–1.25; *P* = 0.037), usual activities (1.14, 1.05–1.24; *P* = 0.001) at the 8-month follow-up (see Supplementary material online, *Figure S6*, *Figure 3*).

At 8 months, sacubitril/valsartan was also associated with greater overall improvement and less worsening in EQ-5D-3L-LSS compared to the active comparator: OR: 1.16 (95%CI: 1.08–1.24) (see Supplementary material online, *Figure S7*). Findings were consistent in individual trials (*Graphical abstract*). Similar findings were also observed irrespective of sex (see Supplementary material online, *Figure S8*). The effect of sacubitril/valsartan on EQ-5D VAS is presented in Supplementary material online, *Table S13*.

### Association between EQ-5D-3L-LSS and clinical outcomes

**Figure 4:**
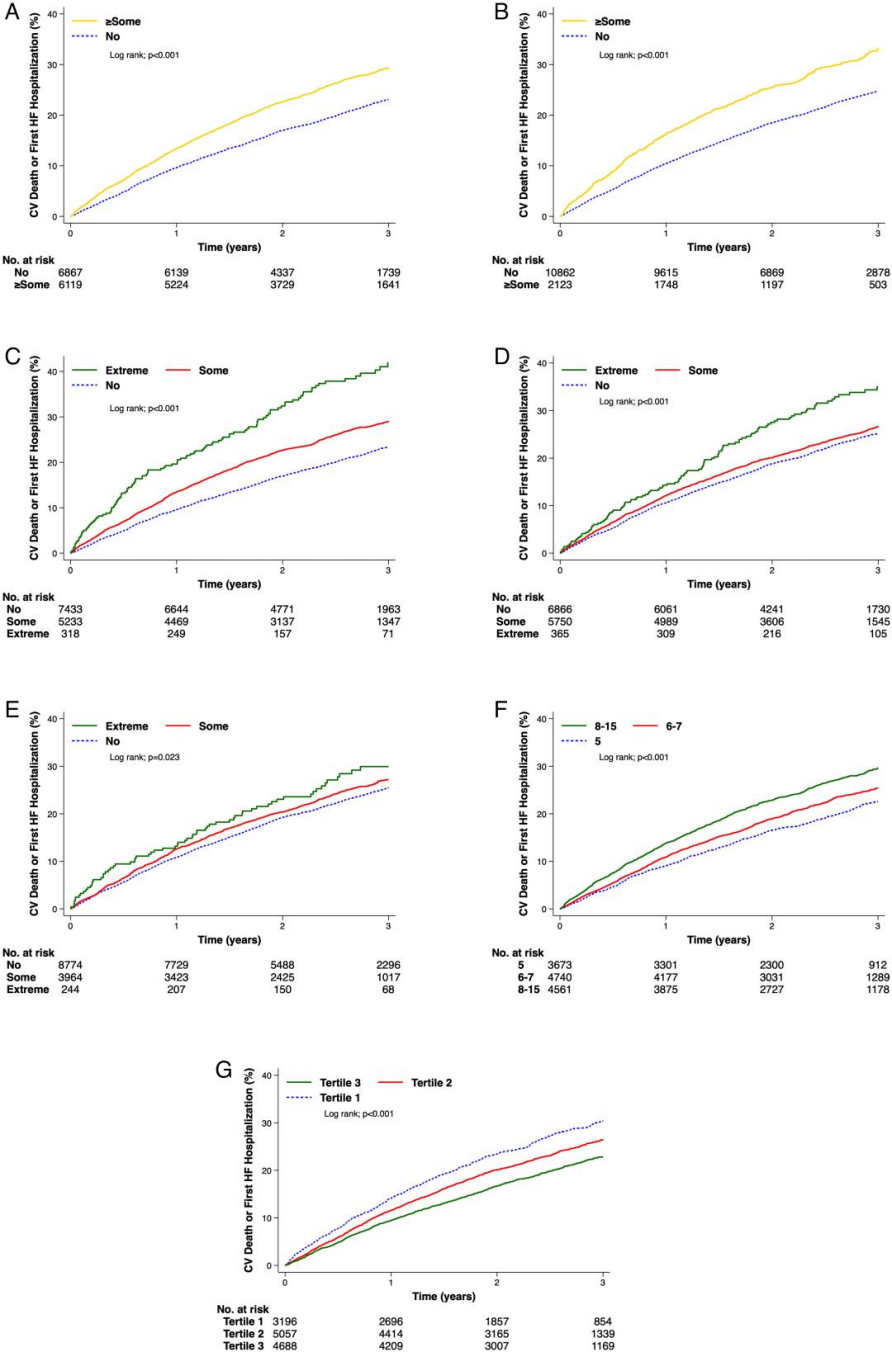
Cumulative incidence of the composite outcome according to baseline EuroQol Five-dimension Three-level (EQ-5D-3L) Questionnaire. (*A*) ‘Mobility’ domain; (*B*) ‘self-care’ domain; (*C*) ‘usual activities’ domain; (*D*) ‘pain/discomfort’ domain; (*E*) ‘anxiety/depression’ domain; (*F*) Level Sum Score (LSS) category divided by tertile; (*G*) Visual Analog Scale (VAS) category divided by tertile. The patients reported ‘Some problem’ and ‘Extreme problem’ were combined into one group due to a small number in the worst category of ‘mobility’ domain (*n* = 27) and ‘self-care’ domain (*n* = 73). CV, cardiovascular; HF, heart failure.

**Figure 5:**
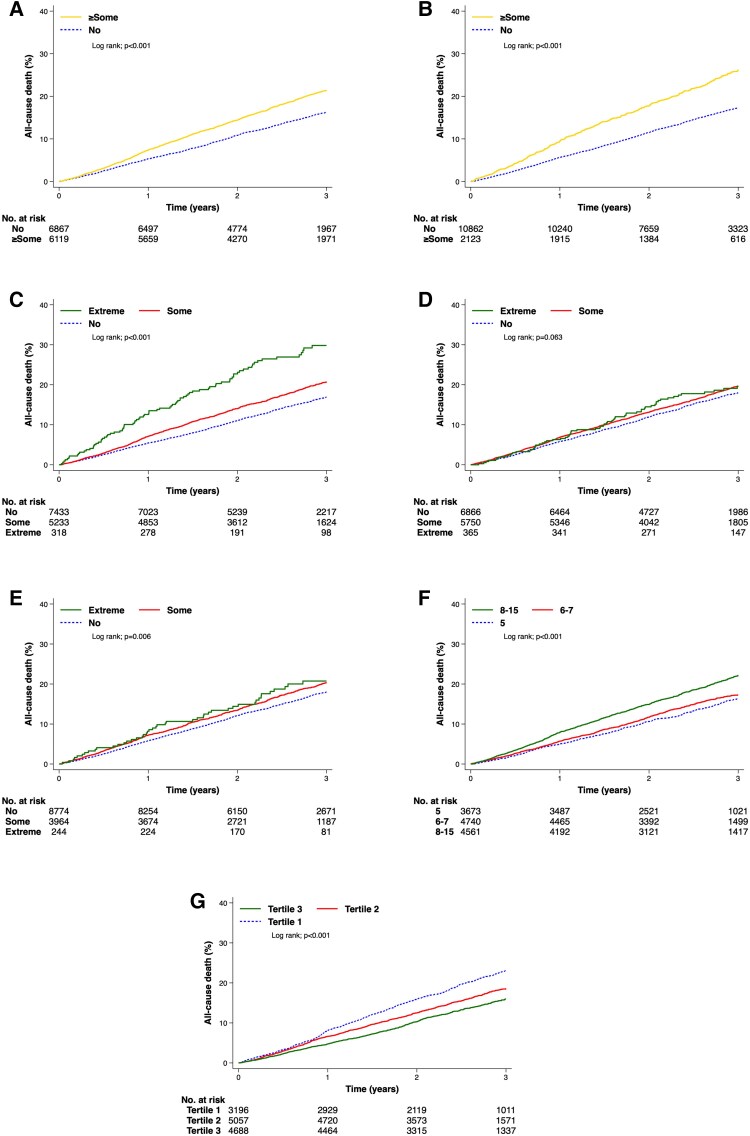
Cumulative incidence of all-cause death according to baseline EuroQol Five-dimension Three-level (EQ-5D-3L) Questionnaire. (*A*) ‘Mobility’ domain; (*B*) ‘self-care’ domain; (*C*) ‘usual activities’ domain; (*D*) ‘pain/discomfort’ domain; (*E*) ‘anxiety/depression’ domain; (*F*) Level Sum Score (LSS) category divided by tertile; (*G*) Visual Analog Scale (VAS) category divided by tertile. The patients reported ‘Some problem’ and ‘Extreme problem’ were combined into one group due to a small number in the worst category of ‘mobility’ domain (*n* = 27) and ‘self-care’ domain (*n* = 73).

The association between EQ-5D-3L-LSS and clinical outcomes is presented in Supplementary material online, *Tables S14*–S16 (overall cohort, HFrEF, and HFmrEF/HFpEF), *Figures 4* and *5* (composite of cardiovascular death or first heart failure hospitalization, and all-cause death), Supplementary material online, *Figures S9* and *S10* (first heart failure hospitalization, and cardiovascular death), and *Graphical abstract*. Patients with higher EQ-5D-3L-LSS (i.e. worse HRQL) had a significantly higher risk of all outcomes evaluated. Compared with patients in the best LSS tertile (T1, LSS = 5), those in T2 (LSS 6–7) had a 21% higher risk of first HF hospitalization or CV death (HR = 1.21; 95%CI: 1.10–1.33), while those in T3 (LSS 8–15) had a 50% higher risk (HR = 1.50; 95%CI: 1.37–1.65), with corresponding rates (95% confidence interval) per 100 person-years for this composite outcome for T1, T2, and T3 of 8.6 (8.0–9.3), 10.0 (9.4–10.6), 12.2 (11.6–12.9), respectively. Although adjustments for prognostic factors, including NT-proBNP, attenuated the hazard ratios, higher EQ-5D-3L-LSS (i.e. worse HRQL) remained significantly associated with worse outcomes after adjustment (see Supplementary material online, *Tables S14*–S16). The association between EQ-5D VAS and the outcomes showed similar results (see Supplementary material online, *Table S17*, *Figures 4* and *5*, and Supplementary material online, *Figures S9* and *S10*). The relationship between EQ-5D-3L-LSS at 8 months and subsequent clinical outcomes is presented in Supplementary material online, *Table S18*.

Associations between individual EQ-5D-3L dimension scores and outcomes revealed a pattern similar to that seen for the overall EQ-5D-3L-LSS (*Figures 4* and *5*, and Supplementary material online, *Figures S9* and *S10*: (A) mobility, (B) self-care, (C) usual activities, (D) pain/discomfort, and (E) anxiety/depression).

Sacubitril/valsartan, compared with a RAS comparator, reduced the risk of the primary outcome across LSS tertiles: T1: HR = 0.87 (95%CI: 0.75–1.00); T2: 0.80 (95%CI: 0.71–0.90); T3: 0.87 (95%CI: 0.77–0.97); *P*_interaction_ = 0.59 (see Supplementary material online, *Tables S19*: overall cohort, *Tables 3* and *4*: HFrEF and HFmrEF/HFpEF). Similar findings were also observed for HF hospitalization, CV death, all-cause death, and total HF hospitalizations and CV death.

**Table 3:** Effect of randomized treatment on outcomes according to baseline EuroQol Five-dimension Three-level (EQ-5D-3L) questionnaire level sum score (LSS) category divided by tertile in HFrEF

	Tertile 1: 5		Tertile 2: 6–7		Tertile 3: 8–15		
	Sacubitril/ valsartan	Active RAS comparator^d^	Sacubitril/ valsartan	Active RAS comparator^d^	Sacubitril/ valsartan	Active RAS comparator^d^	Interaction *P-*Value
*N*	1289	1293	1487	1457	1350	1395	
**CV death or HF hospitalization**							
*N* (%)	245 (19.0)	290 (22.4)	292 (19.6)	392 (26.9)	363 (26.9)	416 (29.8)	
Rate per 100 patient-years (95%CI)	8.9 (7.8–10.1)	10.7 (9.6–12)	9.3 (8.3–10.4)	13.3 (12–14.7)	13.4 (12.1–14.9)	13.9 (12.1–14.9)	
Unadjusted HR (95%CI)^a^	0.83 (0.70–0.98)		0.70 (0.60–0.81)		0.88 (0.76–1.01)		0.10
Adjusted HR (95%CI)^b^	0.81 (0.68–0.96)		0.72 (0.62–0.84)		0.90 (0.78–1.04)		0.10
**First HF hospitalization**							
*N* (%)	136 (10.6)	167 (12.9)	186 (12.5)	243 (16.7)	209 (15.5)	235 (16.9)	
Rate per 100 patient-years (95%CI)	4.9 (4.2–5.8)	6.2 (5.3–7.2)	5.9 (5.1–6.8)	8.2 (7.3–9.3)	7.7 (6.8–8.9)	8.7 (7.6–9.9)	
Unadjusted HR (95%CI)^a^	0.80 (0.63–1.00)		0.71 (0.59–0.86)		0.90 (0.74–1.08)		0.27
Adjusted HR (95%CI)^b^	0.76 (0.61–0.96)		0.74 (0.61–0.89)		0.92 (0.76–1.11)		0.22
**CV death**							
*N* (%)	147 (11.4)	184 (14.2)	166 (11.2)	231 (15.9)	236 (17.5)	268 (19.2)	
Rate per 100 patient-years (95%CI)	5.1 (4.3–6.0)	6.4 (5.5–7.3)	5.0 (4.3–5.8)	7.2 (6.3–8.2)	8.1 (7.1–9.2)	9.0 (8.0–10.1)	
Unadjusted HR (95%CI)^a^	0.80 (0.64–0.99)		0.69 (0.57–0.84)		0.89 (0.75–1.07)		0.16
Adjusted HR (95%CI)^b^	0.80 (0.64–0.99)		0.72 (0.59–0.89)		0.92 (0.77–1.09)		0.17
**Non-CV death**							
*N* (%)	31 (2.4)	24 (1.9)	48 (3.2)	45 (3.1)	38 (2.8)	40 (2.9)	
Rate per 100 patient-years (95%CI)	1.1 (0.8–1.5)	0.8 (0.6–1.2)	1.4 (1.1–1.9)	1.4 (1–1.9)	1.3 (0.9–1.8)	1.3 (1.0–1.8)	
Unadjusted HR (95%CI)^a^	1.28 (0.75–2.17)		1.02 (0.68–1.53)		0.97 (0.62–1.52)		0.71
Adjusted HR (95%CI)^b^	1.21 (0.71–2.08)		1.05 (0.70–1.58)		0.92 (0.58–1.44)		0.78
**All-cause death**							
*N* (%)	190 (14.7)	218 (16.9)	221 (14.9)	287 (19.7)	288 (21.3)	320 (22.9)	
Rate per 100 patient-years (95%CI)	6.6 (5.7–7.6)	7.5 (6.6–8.6)	6.6 (5.8–7.5)	8.9 (8–10)	9.8 (8.8–11)	10.7 (9.6–12.0)	
Unadjusted HR (95%CI)^a^	0.87 (0.72–1.06)		0.74 (0.62–0.88)		0.92 (0.78–1. 70)		0.20
Adjusted HR (95%CI)^b^	0.87 (0.71–1.05)		0.77 (0.65–0.92)		0.93 (0.79–1. 90)		0.26
**Total HF hospitalizations and CV death**							
*N*	349	444	478	642	565	657	
Rate per 100 patient-years (95%CI)	12.0 (10.4–14.0)	15.3 (13.4–17.5)	14.3 (12.4–16.4)	20.0 (17.8–22.5)	19.3 (17.0–21.9)	22.0 (19.7–24.6)	
Unadjusted RR (95%CI)^c^	0.76 (0.60–0.95)		0.64 (0.52–0.79)		0.88 (0.72–1.06)		0.11
Adjusted RR (95%CI)^b^	0.72 (0.58–0.90)		0.63 (0.52–0.77)		0.83 (0.70–1.00)		0.16

BMI, body mass index; CI, confidence interval; CV, cardiovascular; eGFR, estimated glomerular filtration rate; HF, heart failure; HFrEF, heart failure with reduced ejection fraction; hosp. hospitalization; HR, hazard ratio; LVEF, left ventricular ejection fraction; NT-proBNP, N-terminal pro B-type natriuretic peptide; NYHA, New York Heart Association; SBP, systolic blood pressure.

^a^Baseline model stratified by study and study-specific geographic region.

^b^Further adjusted for age, sex, heart rate, SBP, BMI, NYHA functional class III/IV, LVEF, eGFR, NT-proBNP (log-transformed), atrial fibrillation, ischaemic aetiology, myocardial infarction, and stroke.

^c^Adjusted for geographic region and study.

^d^Enalapril or Valsartan.

**Table 4:** Effect of randomized treatment on outcomes according to baseline EuroQol Five-dimension Three-level (EQ-5D-3L) questionnaire level sum score (LSS) category divided by tertile in HFmrEF/HFpEF

	Tertile 1: 5		Tertile 2: 6–7		Tertile 3: 8–15		
	Sacubitril/ valsartan	Active RAS comparator^d^	Sacubitril/ valsartan	Active RAS comparator^d^	Sacubitril/ valsartan	Active RAS comparator^d^	Interaction *P-*Value
*N*	549	542	880	916	930	886	
**CV death or HF hospitalization**							
*N* (%)	95 (17.3)	95 (17.5)	202 (23.0)	203 (22.2)	217 (23.3)	240 (27.1)	
Rate per 100 patient-y ars (95%CI)	6.4 (5.2–7.8)	6.5 (5.4–8.0)	8.5 (7.4–9.8)	8.3 (7.3–9.6)	9.0 (7.9–10.3)	10.6 (9.3–12.0)	
Unadjusted HR (95%CI)^a^	0.98 (0.74–1.30)		1.00 (0.82–1.22)		0.85 (0.70–1.02)		0.41
Adjusted HR (95%CI)^b^	1.00 (0.75–1.34)		0.95 (0.78–1.16)		0.84 (0.70–1.01)		0.50
**First HF hospitalization**							
*N* (%)	76 (13.8)	79 (14.6)	162 (18.4)	149 (16.3)	157 (16.9)	187 (21.1)	
Rate per 100 patient-years (95%CI)	5.1 (4.1–6.4)	5.4 (4.4–6.8)	6.8 (5.9–8)	6.1 (5.2–7.2)	6.5 (5.6–7.6)	8.2 (7.1–9.5)	
Unadjusted HR (95%CI)^a^	0.95 (0.69–1.30)		1.09 (0.87–1.36)		0.78 (0.63–0.97)		0.10
Adjusted HR (95%CI)^b^	0.97 (0.70–1.33)		1.05 (0.83–1.31)		0.79 (0.64–0.98)		0.18
**CV death**							
*N* (%)	32 (5.8)	25 (4.6)	74 (8.4)	94 (9.9)	95 (10.2)	93 (10.5)	
Rate per 100 patient-years (95%CI)	2.0 (1.4–2.8)	1.6 (1.1–2.4)	2.8 (2.3–3.6)	3.4 (2.8–4.2)	3.6 (3–4.4)	3.6 (3.0–4.5)	
Unadjusted HR (95%CI)^a^	1.20 (0.71–2.03)		0.82 (0.60–1.11)		1.01 (0.76–1.34)		0.36
Adjusted HR (95%CI)^b^	1.25 (0.73–2.13)		0.76 (0.55–1.03)		0.99 (0.74–1.32)		0.18
**Non-CV death**							
*N* (%)	23 (4.2)	31 (5.7)	24 (2.7)	30 (3.3)	53 (5.7)	54 (6.1)	
Rate per 100 patient-years (95%CI)	1.4 (1.0–2.2)	2.0 (1.4–2.8)	0.9 (0.6–1.4)	1.1 (0.8–1.6)	2.0 (1.5–2.6)	2.1 (1.6–2.8)	
Unadjusted HR (95%CI)^a^	0.73 (0.42–1.25)		0.81 (0.41–1.39)		0.96 (0.66–1.40)		0.70
Adjusted HR (95%CI)^b^	0.67 (0.38–1.17)		0.75 (0.44–1.29)		0.93 (0.63–1.37)		0.71
**All-cause death**							
*N* (%)	61 (11.1)	58 (10.7)	112 (12.7)	131 (14.3)	165 (17.7)	152 (17.2)	
Rate per 100 patient-years (95%CI)	3.8 (3.0–4.9)	3.7 (2.9–4.8)	4.3 (3.6–5.2)	5.0 (4.2–5.9)	6.3 (5.4–7.3)	5.9 (5.1–7.0)	
Unadjusted HR (95%CI)^a^	1.01 (0.70–1.44)		0.86 (0.67–1.11)		1.07 (0.86–1.33)		0.46
Adjusted HR (95%CI)^b^	0.99 (0.69–1.44)		0.82 (0.63–1.06)		1.06 (0.84–1.32)		0.35
**Total HF hospitalizations and CV death**							
*N*	170	158	342	367	363	446	
Rate per 100 patient-years (95%CI)	10.7 (8.3–13.6)	10.1 (7.9–12.9)	13.2 (11.1–15.6)	13.9 (11.7–16.5)	13.8 (11.9–16.0)	17.4 (14.8–20.5)	
Unadjusted RR (95%CI)^c^	1.10 (0.76–1.57)		0.84 (0.65–1.09)		0.82 (0.65–1.05)		0.40
Adjusted RR (95%CI)^b^	1.19 (0.84–1.68)		0.88 (0.69–1.12)		0.81 (0.64–1.03)		0.21

BMI, body mass index; CI, confidence interval; CV, cardiovascular; eGFR, estimated glomerular filtration rate; HF, heart failure; HFmrEF, heart failure with mildly reduced ejection fraction; HFpEF, heart failure with preserved ejection fraction; hosp. hospitalization; HR, hazard ratio; LVEF, left ventricular ejection fraction; NT-proBNP, N-terminal pro B-type natriuretic peptide; NYHA, New York Heart Association; SBP, systolic blood pressure.

^a^Baseline model stratified by study and study-specific geographic region.

^b^Further adjusted for age, sex, heart rate, SBP, BMI, NYHA functional class III/IV, LVEF, eGFR, NT-proBNP (log-transformed), atrial fibrillation, ischaemic aetiology, myocardial infarction, and stroke.

^c^Adjusted for geographic region and study.

^d^Enalapril or Valsartan.

## Discussion

In this pooled analysis of PARADIGM-HF and PARAGON-HF, we found that higher EQ-5D-3L-LSS (i.e. worse HRQL) was associated with a greater risk of HF-related hospitalizations and mortality. Importantly, treatment with sacubitril/valsartan improved patient-reported functioning (reflected by the mobility, self-care, and usual activities dimensions of EQ-5D) in patients with HFrEF and HFmrEF/HFpEF. To our knowledge, this study represents the first large-scale investigation evaluating the impact of sacubitril/valsartan on both the overall EQ-5D-3L-LSS and EQ-5D-3L dimension-specific scores in patients with HFrEF and HFmrEF/HFpEF. These findings highlight the potential clinical utility of EQ-5D as a tool for assessing HRQL in HF and evaluating the effectiveness of HF treatments.

The relationship between EQ-5D-3L-LSS and clinical outcomes aligns with prior studies demonstrating a correlation between EQ-5D scores and disease-specific HRQL measures, including the Minnesota Living with Heart Failure Questionnaire (MLHFQ), KCCQ, and NYHA functional class.^19,24-28^ Patients with worse baseline EQ-5D-3L-LSS had a significantly greater risk of adverse HF outcomes, even after adjusting for established prognostic markers such as NT-proBNP levels. A similar pattern was also observed across individual domain scores and the VAS. This reinforces the potential usefulness of incorporating HRQL assessments into routine clinical practice, as they provide valuable prognostic information beyond traditional biomarkers and clinical variables.^20^ Furthermore, EQ-5D provides a broader, generic assessment of patient well-being and enables cross-disease evaluations as it is not a HF-specific tool.^1,29^

Contrary to expectation, NT-proBNP levels were similar across tertiles of baseline EQ-5D-3L-LSS in HFmrEF/HFpEF. This suggests that the EQ-5D may capture aspects of health status, including physical functioning, symptoms (pain/discomfort), and mental well-being, that are not fully reflected by traditional biomarkers such as natriuretic peptides. This further reinforces the potential value of integrating patient-reported outcomes into clinical assessment, particularly in patients with HFmrEF/HFpEF. On the other hand, as expected, we did observe a significant trend in NT-proBNP levels across EQ-5D categories in DAPA-HF and DELIVER trials. The discrepancies between those findings and ours may be explained by differences in trial design: (i) the presence of a run-in period in PARADIGM-HF and PARAGON-HF; (ii) the timing of NT-proBNP measurement at screening rather than at randomization; and (iii) the use of an active comparator rather than a placebo. Additionally, differences in the EQ-5D instrument versions used (EQ-5D-3L vs. EQ-5D-5L) may also have contributed to the observed variation.

The prevalence of problems (‘some’ or ‘extreme’) across individual EQ-5D was mobility 47%, self-care 16%, usual activities 43%, pain/discomfort 47%, and anxiety/depression 32%. In DAPA-HF and DELIVER trials, higher proportions of participants reported problems in all EQ-5D (63%, 33%, 60%, 55%, and 43% in mobility, self-care, usual activities, pain/discomfort, and anxiety/depression, respectively).^17^ This may reflect the use of the EQ-5D-5L in DAPA-HF/DELIVER, rather than the EQ-5D-3L in PARADIGM-HF/PARAGON-HF. The EQ-5D-5L was introduced later to enhance measurement precision and reduce ceiling and floor effects observed with the EQ-5D-3L, and our findings support the view that the EQ-5D-5L may have greater sensitivity.^30^ Moreover, participants in the DAPA-HF trial exhibited poorer baseline health status, as indicated by lower KCCQ scores and less favourable prognostic profiles. While overall EQ-5D-3L-LSS were broadly similar between patients with HFrEF and HFmrEF/HFpEF, domain-specific differences were observed. Notably, patients with HFmrEF/HFpEF more frequently reported impairments in mobility and experienced greater levels of pain or discomfort than those with HFrEF. These findings align with the demographic and clinical profile of patients with HFpEF, who are often older, more likely to be women, and have a higher burden of comorbidities such as obesity, arthritis, or musculoskeletal disorders that may impact physical function and pain perception. These observations highlight the importance of considering domain-specific health status in addition to summary scores, particularly when evaluating patients with different HF phenotypes.

Consistent with other studies, we found high proportions of patients experiencing pain/discomfort and anxiety/depression, domains that differ from disease-specific instruments and which are often underrecognized in HF. Few studies have investigated pain in outpatients with HF. Pain may stem from comorbid conditions rather than HF *per se*.^17^ Interestingly, pain/discomfort was the only dimension that did not improve with sacubitril/valsartan or dapagliflozin, suggesting that HF therapies may not directly address this domain.^17^ Patients reporting greater pain were also more likely to have multiple comorbid conditions, a trend consistent with these individuals exhibiting worse scores across other EQ-5D-3L dimensions. Moreover, more severe pain was associated with more advanced heart failure, as reflected by a higher NYHA functional class. Unfortunately, none of the trials included captured specific concomitant conditions that may contribute directly to chronic pain, such as osteoarthritis, spinal disorders, connective tissue diseases, or chronic inflammatory conditions. The pain-related burden in HF patients warrants further exploration. Beyond the influence of comorbidities, some evidence suggests that HF itself may exacerbate pain perception through central sensitization due to heightened nociceptive signalling and increased pain sensitivity, potentially amplifying discomfort from underlying diseases.^31^ This neurobiological dysregulation may also explain why some HF patients report debilitating pain, even in the absence of significant musculoskeletal abnormalities. On the other hand, self-reported pain may reflect broader emotional distress or negative affect, rather than specific physical pathology, and responses can be shaped by general pessimism and unhappiness rather than careful symptom appraisal.

Additionally (and perhaps related), psychological distress is another underappreciated component of HRQL impairment in HF. In the Swedish Heart Failure Registry, 40% of patients with HFrEF and 44% of those with HFpEF reported experiencing some degree of anxiety or depression, as also assessed by EQ-5D-3L.^32^ In the current study, it was 32% and 33% in HFrEF and HFmrEF/HFpEF, respectively. This difference may reflect the selected nature of patients enrolled in trials or might be attributed to more comprehensive clinical management and closer patient follow-up in our trial populations, underscoring the potential impact of structured care on mental health outcomes in HF patients.

Sacubitril/valsartan improved EQ-5D-3L-LSS over 8 months, with patients receiving this treatment experiencing greater improvement (21.3% vs. 19.9%) and less worsening (19.0% vs. 21.7%) compared to a RAS blocker. This effect was consistent irrespective of HF phenotype or sex. Treatment benefit was also observed on mobility, self-care, and usual activities domains, suggesting that sacubitril/valsartan contributes to functional improvements in daily living. These findings also align with improvements in the disease-specific KCCQ with sacubitril/valsartan.^33,34^ Our study extends these observations to the generic EQ-5D-3L, reinforcing its applicability across diverse HF populations. In addition to the present results with sacubitril/valsartan, other trials assessing HRQL in HF, such as DAPA-HF and DELIVER, have also shown a beneficial effect of dapagliflozin in improving EQ-5D-LSS, VAS, and utility index score.^16,17^

Detailed analysis of individual EQ-5D-3L dimensions provides insights into which aspects of patient well-being are most impacted by HF and which are most responsive to treatment. Compared with the comparator, sacubitril/valsartan significantly improved functional domains (mobility, self-care, and usual activities), which are essential for independent living and overall well-being. This is consistent with previous findings from PARADIGM-HF, where sacubitril/valsartan enhanced KCCQ physical and social limitation scores.^33,35^ Similarly, in the CARE-HF study, CRT was associated with a significant improvement in disease-specific quality of life (Minnesota Living with Heart Failure) at 18 months, with a mean between-group difference of 10.7 points (95% CI: 7.6–13.8), primarily driven by enhanced physical functioning.^18^ Although the change in anxiety/depression was directionally similar, it was of borderline statistical significance. This domain did demonstrate a significant improvement in a patient-level meta-analysis of DAPA-HF and DELIVER and the discrepancy between these analyses may again reflect the use of the EQ-5D-3L vs. EQ-5D-5L.^17^

By contrast, pain/discomfort showed only a small and non-significant change with HF therapy, consistent with the analyses of DAPA-HF/DELIVER.

### Limitations

This study has similar limitations to other analyses of the EQ-5D-LSS.^17^ Although there are always questions about response bias and potential differences between responders and non-responders, the response rate to the questionnaire was high (98.3% at baseline and 90.3% at 8 months). This study focused on sacubitril/valsartan, so findings may not apply to other HF therapies, and confounding from comorbidities could have influenced the results.

### Conclusions

Both the overall EQ-5D-3L-LSS and its individual dimension scores were correlated with clinical characteristics and outcomes. Treatment with sacubitril/valsartan led to reductions in HF-related events across the tertiles of baseline EQ-5D-3L-LSS in patients with both HFrEF and HFmrEF/HFpEF. Additionally, improvements were observed across multiple EQ-5D, particularly in mobility, self-care, and usual activities, and overall HRQL in patients receiving sacubitril/valsartan.

## Supplementary Material

pvaf064_Supplementary_Data

## Data Availability

The data underlying this article will be shared on reasonable request to the corresponding author.
