# Supplementary material for: Sacubitril/valsartan and quality of life assessed using the EuroQol Five-dimension Three-level questionnaire level sum score (EQ-5D-3L-LSS) in patients with HFrEF and HFmrEF/HFpEF
Source: Eur Heart J Cardiovasc Pharmacother. 2025 Aug 21;11(7):574–89. doi: 10.1093/ehjcvp/pvaf064 (PMC12582660; doi:10.1093/ehjcvp/pvaf064)

**Sacubitril/valsartan and Quality of Life Assessed Using the EuroQol 5-Dimension 3-Level Questionnaire Level Sum Score (EQ-5D-3L-LSS) in Patients with HFrEF and HFmrEF/HFpEF**

Supplementary Material

| **Methods** | Page |
| --- | --- |
| Trials and patients | 4 |
|  |  |
| **Tables** |  |
| Supplementary Table 1: Patients completed the instrument in the overall cohort of PADADIGM-HF and PARAGON-HF | 6 |
| Supplementary Table 2: Patients completed the EuroQol 5-Dimension 3-Level (EQ-5D-3L) health questionnaire according to answers in PADADIGM-HF | 7 |
| Supplementary Table 3: Patients completed the EuroQol 5-Dimension 3-Level (EQ-5D-3L) health questionnaire according to answers in PARAGON-HF | 8 |
| Supplementary Table 4: Baseline characteristics according to baseline EuroQol 5-Dimension 3-Level (EQ-5D-3L) questionnaire Level Sum Score (LSS) category divided by tertile | 9 |
| Supplementary Table 5: Detailed baseline characteristics according to baseline EuroQol 5-Dimension 3-Level (EQ-5D-3L) questionnaire Level Sum Score (LSS) category divided by tertile | 12 |
| Supplementary Table 6: Baseline characteristics according to baseline EuroQol 5-Dimension 3-Level (EQ-5D-3L) questionnaire Visual Analog Scale (VAS) category divided by tertile | 19 |
| Supplementary Table 7: Patients completed the instrument in the cohort of PARADIGM-HF (HFrEF) and PARAGON-HF (HFpEF) at baseline | 21 |
| Supplementary Table 8: Baseline characteristics based on answers to the EuroQol 5-Dimension 3-Level (EQ-5D-3L) “mobility” question | 22 |
| Supplementary Table 9: Baseline characteristics based on answers to the EuroQol 5-Dimension 3-Level (EQ-5D-3L) “self-care” question | 24 |
| Supplementary Table 10: Baseline characteristics based on answers to the EuroQol 5-Dimension 3-Level (EQ-5D-3L) “usual activities” question | 26 |
| Supplementary Table 11: Baseline characteristics based on answers to the EuroQol 5-Dimension 3-Level (EQ-5D-3L) “pain/discomfort” question | 28 |
| Supplementary Table 12: Baseline characteristics based on answers to the E EuroQol 5-Dimension 3-Level (EQ-5D-3L) “anxiety/depression” question | 30 |
| Supplementary Table 13: Effect of sacubitril/valsartan versus active comparator on EuroQol 5-Dimension 3-Level (EQ-5D-3L) questionnaire Visual Analog Scale (VAS) over time (8 months) | 32 |
| Supplementary Table 14: Clinical outcomes according to baseline EuroQol 5-Dimension 3-Level (EQ-5D-3L) Level Sum Score (LSS) category divided by tertile | 33 |
| Supplementary Table 15: Clinical outcomes according to baseline EuroQol 5-Dimension 3-Level (EQ-5D-3L) Level Sum Score (LSS) category divided by tertile in HFrEF | 35 |
| Supplementary Table 16: Clinical outcomes according to baseline EuroQol 5-Dimension 3-Level (EQ-5D-3L) Level Sum Score (LSS) category divided by tertile in HFmrEF/HFpEF | 37 |
| Supplementary Table 17: Clinical outcomes according to baseline EuroQol 5-Dimension 3-Level (EQ-5D-3L) questionnaire Visual Analog Scale (VAS) category divided by tertile | 39 |
| Supplementary Table 18: Association of 8-month EuroQol 5-Dimension 3-Level (EQ-5D-3L) questionnaire Level Sum Score (LSS) with clinical outcomes | 41 |
| Supplementary Table 19: Effect of randomized treatment on outcomes according to baseline EuroQol 5-Dimension 3-Level (EQ-5D-3L) questionnaire Level Sum Score (LSS) category divided by tertile | 43 |
|  |  |
| **Figures** |  |
| Supplementary Figure 1: Distribution of answers to each question of the EuroQol 5-Dimension 3-Level (EQ-5D-3L) health questionnaire | 46 |
| Supplementary Figure 2: Distribution of EuroQol 5-Dimension 3-Level (EQ-5D-3L) Level Sum Score (LSS) | 47 |
| Supplementary Figure 3: Distribution and changes in answers to the EuroQol 5-Dimension 3-Level (EQ-5D-3L) “mobility” question from baseline to 8 months follow-up | 48 |
| Supplementary Figure 4: Distribution and changes in answers to the EuroQol 5-Dimension 3-Level (EQ-5D-3L) “self-care” question from baseline to 8 months follow-up | 49 |
| Supplementary Figure 5: Distribution and changes in answers to the EuroQol 5-Dimension 3-Level (EQ-5D-3L) “usual activities” question from baseline to 8 months follow-up | 50 |
| Supplementary Figure 6**:** Effect of sacubitril/valsartan versus active comparator on EuroQol 5-Dimension 3-Level (EQ-5D-3L) questionnaire dimension over time (8 months) | 51 |
| Supplementary Figure 7: Effect of Sacubitril/Valsartan versus active comparator on EuroQol 5-Dimension 3-Level (EQ-5D-3L) Level Sum Score (LSS) over time (8 months) in patients with HF | 52 |
| Supplementary Figure 8: Effect of Sacubitril/Valsartan versus active comparator on EuroQol 5-Dimension 3-Level (EQ-5D-3L) Level Sum Score (LSS) over time (8 months) by sex | 53 |
| Supplementary Figure 9: Cumulative incidence of first heart failure hospitalization according to baseline EuroQol 5-Dimension 3-Level (EQ-5D-3L) questionnaire | 54 |
| Supplementary **F**igure 10: Cumulative incidence of cardiovascular death according to baseline EuroQol 5-Dimension 3-Level (EQ-5D-3L) questionnaire | 55 |

**Trials and patients**

To ascertain participants' tolerability to the study medications, both trials implemented sequential single-blind run-in periods before randomization. In PARADIGM-HF, patients were up-titrated to the target doses of both enalapril and sacubitril/valsartan, whereas in PARAGON-HF, patients were stabilized at half-target doses before randomization. Only participants who tolerated both medications were eligible for randomization.

The inclusion criteria for both trials were broadly comparable, with key differences in age (≥18/ ≥50 years for PARADIGM-HF/PARAGON-HF) and LVEF thresholds (≤40%/≥45% for PARADIGM-HF/PARAGON-HF). Both trials required participants to have signs or symptoms of HF (New York Heart Association [NYHA] functional classes II-IV), and have elevated concentrations of B-type natriuretic peptide (BNP, pg/mL) or N-terminal proBNP (NT-proBNP, pg/mL) (PARADIGM-HF: BNP ≥ 150/NT-proBNP≥600, ≥100/≥400 if hospitalized in the previous year; PARAGON-HF: NT-proBNP>300 or >900 [if in atrial fibrillation on screening electrocardiogram], >200 or >600 [for atrial fibrillation] if hospitalized for HF within 9 months). Moreover, in PARAGON-HF, patients were required to have evidence of structural heart disease, characterized by left atrial (LA) enlargement or left ventricular (LV) hypertrophy. The exclusion criteria for both studies were similar and included an estimated glomerular filtration rate (eGFR) of < 30 mL/min/1.73m^2^ or serum potassium > 5.2mmol/L at screening.

Supplementary Table 1: Patients completed the instrument in the overall cohort of PADADIGM-HF and PARAGON-HF.

| EQ-5D-3L | Baseline | | | 8 months | | |
| --- | --- | --- | --- | --- | --- | --- |
| Mobility | 12986 | 1 | 6867 | 11925 | 1 | 6327 |
|  |  | 2 | 6092 |  | 2 | 5549 |
|  |  | 3 | 27 |  | 3 | 49 |
| Self-care | 12985 | 1 | 10862 | 11925 | 1 | 9830 |
|  |  | 2 | 2050 |  | 2 | 2011 |
|  |  | 3 | 73 |  | 3 | 84 |
| Usual activities | 12984 | 1 | 7433 | 11924 | 1 | 6819 |
|  |  | 2 | 5233 |  | 2 | 4781 |
|  |  | 3 | 318 |  | 3 | 324 |
| Pain/discomfort | 12981 | 1 | 6866 | 11921 | 1 | 6322 |
|  |  | 2 | 5750 |  | 2 | 5258 |
|  |  | 3 | 365 |  | 3 | 341 |
| Anxiety/depression | 12982 | 1 | 8774 | 11921 | 1 | 8002 |
|  |  | 2 | 3964 |  | 2 | 3676 |
|  |  | 3 | 244 |  | 3 | 243 |
| Level sum score | 12974 | 5 | 3673 | 11915 | 5 | 3615 |
|  |  | 6-7 | 4740 |  | 6-7 | 4033 |
|  |  | 8-15 | 4561 |  | 8-15 | 4267 |

1 = no problem; 2 = some problem; 3 = extreme problem.

EQ-5D-3L, EuroQol 5-Dimension 3-Level questionnaire. PARADIGM-HF, Prospective Comparison of ARNI with ACEI to Determine Impact on Global Mortality and Morbidity in Heart Failure; PARAGON-HF, Prospective Comparison of ARNI with ARB Global Outcomes in HF With Preserved Ejection Fraction.

Supplementary Table 2: Patients completed the EuroQol 5-Dimension 3-Level (EQ-5D-3L) health questionnaire according to answers in PADADIGM-HF.

| EQ-5D-3L | Baseline | | | 8 months | | |
| --- | --- | --- | --- | --- | --- | --- |
| Mobility | 8279 | 1 | 4689 | 7510 | 1 | 4321 |
|  |  | 2 | 3571 |  | 2 | 3155 |
|  |  | 3 | 19 |  | 3 | 34 |
| Self-care | 8278 | 1 | 6929 | 7510 | 1 | 6267 |
|  |  | 2 | 1317 |  | 2 | 1198 |
|  |  | 3 | 32 |  | 3 | 45 |
| Usual activities | 8277 | 1 | 4835 | 7510 | 1 | 4465 |
|  |  | 2 | 3238 |  | 2 | 2861 |
|  |  | 3 | 204 |  | 3 | 184 |
| Pain/discomfort | 8276 | 1 | 4645 | 7507 | 1 | 4242 |
|  |  | 2 | 3471 |  | 2 | 3106 |
|  |  | 3 | 160 |  | 3 | 159 |
| Anxiety/depression | 8277 | 1 | 5591 | 7507 | 1 | 5060 |
|  |  | 2 | 2534 |  | 2 | 2299 |
|  |  | 3 | 152 |  | 3 | 148 |
| Level sum score | 8271 | 5 | 2582 | 7505 | 5 | 2540 |
|  |  | 6-7 | 2944 |  | 6-7 | 2492 |
|  |  | 8-15 | 2745 |  | 8-15 | 2473 |

1 = no problem; 2 = some problem; 3 = extreme problem.

EQ-5D-3L, EuroQol 5-Dimension 3-Level questionnaire. PARADIGM-HF, Prospective Comparison of ARNI with ACEI to Determine Impact on Global Mortality and Morbidity in Heart Failure.

Supplementary Table 3: Patients completed the EuroQol 5-Dimension 3-Level (EQ-5D-3L) health questionnaire according to answers in PARAGON-HF.

| EQ-5D-3L | Baseline | | | 8 months | | |
| --- | --- | --- | --- | --- | --- | --- |
| Mobility | 4707 | 1 | 2178 | 4415 | 1 | 2006 |
|  |  | 2 | 2521 |  | 2 | 2394 |
|  |  | 3 | 8 |  | 3 | 15 |
| Self-care | 4707 | 1 | 3933 | 4415 | 1 | 3563 |
|  |  | 2 | 733 |  | 2 | 813 |
|  |  | 3 | 41 |  | 3 | 39 |
| Usual activities | 4707 | 1 | 2598 | 4414 | 1 | 2354 |
|  |  | 2 | 1995 |  | 2 | 1920 |
|  |  | 3 | 114 |  | 3 | 140 |
| Pain/discomfort | 4705 | 1 | 2221 | 4414 | 1 | 2080 |
|  |  | 2 | 2279 |  | 2 | 2152 |
|  |  | 3 | 205 |  | 3 | 182 |
| Anxiety/depression | 4705 | 1 | 3183 | 4414 | 1 | 2942 |
|  |  | 2 | 1430 |  | 2 | 1377 |
|  |  | 3 | 92 |  | 3 | 95 |
| Level sum score | 4703 | 5 | 1091 | 4410 | 5 | 1075 |
|  |  | 6-7 | 1796 |  | 6-7 | 1541 |
|  |  | 8-15 | 1816 |  | 8-15 | 1794 |

1 = no problem; 2 = some problem; 3 = extreme problem.

EQ-5D-3L, EuroQol 5-Dimension 3-Level questionnaire. PARAGON-HF, Prospective Comparison of ARNI with ARB Global Outcomes in HF With Preserved Ejection Fraction.

Supplementary Table 4: Baseline characteristics according to baseline EuroQol 5-Dimension 3-Level (EQ-5D-3L) questionnaire Level Sum Score (LSS) category divided by tertile. A more detailed list is presented as **Supplementary Table 5**.

|  | **Tertile 1: 5** | **Tertile 2: 6-7** | **Tertile 3: 8-15** | ***P-*Value for trend** |
| --- | --- | --- | --- | --- |
| N, (%) | 3673 (28.3) | 4740 (36.5) | 4561 (35.2) |  |
| HFrEF | 2582 (31.2) | 2944 (35.6) | 2745 (33.2) |  |
| HFmrEF/HFpEF | 1091 (23.2) | 1796 (38.2) | 1816 (38.6) |  |
| Age, yr | 66 (58-74) | 68 (60-76) | 69 (61-77) | <0.001 |
| Female | 810 (22.1) | 1478 (31.2) | 1927 (42.2) | <0.001 |
| Race |  |  |  | <0.001 |
| White | 2282 (62.1) | 3359 (70.9) | 3671 (80.5) |  |
| Black | 164 (4.5) | 205 (4.3) | 159 (3.5) |  |
| Asian | 856 (23.3) | 742 (15.7) | 445 (9.8) |  |
| Others | 371 (10.1) | 434 (9.2) | 286 (6.3) |  |
| SBP, mmHg | 121 (110-133) | 123 (110-135) | 125 (115-136) | <0.001 |
| BMI, kg/m^2^ | 27.5 (24.4-31.0) | 28.2 (25.1-32.0) | 29.4 (25.8-33.6) | <0.001 |
| Atrial fibrillation (history) | 1406 (38.3) | 1970 (41.6) | 2206 (48.4) | <0.001 |
| MI | 1240 (33.8) | 1679 (35.4) | 1716 (37.6) | <0.001 |
| Stroke | 249 (6.8) | 414 (8.7) | 550 (12.1) | <0.001 |
| Diabetes mellitus | 1220 (33.2) | 1770 (37.3) | 1875 (41.1) | <0.001 |
|  |  |  |  |  |
| Time since HF diagnosis |  |  |  | <0.001 |
| $\leq$1yr | 1397 (38.1) | 1630 (34.4) | 1370 (30.1) |  |
| >1-5yrs | 1318 (35.9) | 1731 (36.5) | 1788 (39.3) |  |
| >5yrs | 956 (26.0) | 1377 (29.1) | 1396 (30.7) |  |
| Previous hospitalization for HF | 2061 (56.1) | 2696 (56.9) | 2680 (58.8) | 0.014 |
| NYHA III/IV | 372 (10.1) | 904 (19.1) | 1709 (37.5) | <0.001 |
| KCCQ clinical summary score | 92 (84-98) | 80 (70-90) | 60 (47-73) | <0.001 |
| NT-proBNP, pg/ml | 1282 (724-2399) | 1300 (701-2566) | 1343 (688-2530) | 0.45 |
| LVEF, % | 34 (28-49) | 35 (29-54) | 35 (30-55) | <0.001 |
| Creatinine, μmol/L | 95 (80-112) | 95 (80-114) | 93 (78-112) | 0.013 |
| eGFR, mL/min/1.73m^2^ | 67 (54-80) | 64 (51-77) | 63 (50-76) | <0.001 |
| Diuretics | 3005 (81.8) | 4068 (85.8) | 4056 (88.9) | <0.001 |
| Beta-blocker | 3280 (89.3) | 4185 (88.3) | 3985 (87.4) | 0.007 |
| MRA | 1732 (47.2) | 2040 (43.0) | 2053 (45.0) | 0.08 |
| Pacemaker | 371 (10.1) | 574 (12.1) | 575 (12.6) | <0.001 |
| ICD# | 381 (10.4) | 482 (10.2) | 380 (8.3) | 0.001 |

Data are presented as median (25%-75% quartile) for continuous measures, and n (%) for categorical measures.

# Including CRT-D.

BMI, body mass index; CRT-D, cardiac resynchronization therapy with defibrillator; eGFR, estimated glomerular filtration rate; HF, heart failure; ICD, implantable cardioverter defibrillator; KCCQ, Kansas City Cardiomyopathy Questionnaire; LVEF, left ventricular ejection fraction; MI, myocardial infarction; MRA, mineralocorticoid receptor antagonist; NT-proBNP, N-terminal pro B-type natriuretic peptide; NYHA, New York Heart Association; SBP, systolic blood pressure; yr, year.

Supplementary Table 5: Detailed baseline characteristics according to baseline EuroQol 5-Dimension 3-Level (EQ-5D-3L) questionnaire Level Sum Score (LSS) category divided by tertile

|  | **Tertile 1: 5** | **Tertile 2: 6-7** | **Tertile 3: 8-15** | ***P* Value for trend** |
| --- | --- | --- | --- | --- |
| N, (%) | 3673 (28.3) | 4740 (36.5) | 4561 (35.2) |  |
| HFrEF | 2582 (31.2) | 2944 (35.6) | 2745 (33.2) |  |
| HFmrEF/HFpEF | 1091 (23.2) | 1796 (38.2) | 1816 (38.6) |  |
| **Demographic characteristics** |  |  |  |  |
| Age, yr | 66 (58-74) | 68 (60-76) | 69 (61-77) | <0.001 |
| Gender |  |  |  | <0.001 |
| Women | 810 (22.1) | 1478 (31.2) | 1927 (42.2) |  |
| Men | 2863 (77.9) | 3262 (68.8) | 2634 (57.8) |  |
| Region |  |  |  | <0.001 |
| North America | 315 (8.6) | 433 (9.1) | 410 (9.0) |  |
| Latin America | 628 (17.1) | 700 (14.8) | 466 (10.2) |  |
| Western Europe | 994 (27.1) | 1269 (26.8) | 1086 (23.8) |  |
| Eastern Europe† | 856 (23.3) | 1568 (33.1) | 2085 (45.7) |  |
| Asia/Pacific and other | 880 (24.0) | 770 (16.2) | 514 (11.3) |  |
| Race |  |  |  | <0.001 |
| White | 2282 (62.1) | 3359 (70.9) | 3671 (80.5) |  |
| Black | 164 (4.5) | 205 (4.3) | 159 (3.5) |  |
| Asian | 856 (23.3) | 742 (15.7) | 445 (9.8) |  |
| Others | 371 (10.1) | 434 (9.2) | 286 (6.3) |  |
| SBP, mmHg | 121 (110-133) | 123 (110-135) | 125 (115-136) | <0.001 |
| SBP category |  |  |  | <0.001 |
| <110 mmHg | 646 (17.6) | 793 (16.7) | 588 (12.9) |  |
| 110-119 mmHg | 830 (22.6) | 986 (20.8) | 904 (19.8) |  |
| 120-129 mmHg | 872 (23.7) | 1093 (23.1) | 1143 (25.1) |  |
| 130-139 mmHg | 685 (18.6) | 939 (19.8) | 968 (21.2) |  |
| ≥140 mmHg | 640 (17.4) | 929 (19.6) | 957 (21.0) |  |
| DBP, mmHg | 73 (67-80) | 73 (67-80) | 75 (68-80) | <0.001 |
| PP, mmHg | 49 (40-58) | 50 (40-60) | 50 (40-60) | <0.001 |
| MAP, mmHg | 90 (83-97) | 90 (83-97) | 927 (84-99) | <0.001 |
| HR, bpm | 70 (62-78) | 70 (63-78) | 71 (64-80) | <0.001 |
| BMI, kg/m^2^ | 27.5 (24.4-31.0) | 28.2 (25.1-32.0) | 29.4 (25.8-33.6) | <0.001 |
| Weight category |  |  |  | <0.001 |
| <18.5 kg/m^2^ | 52 (1.4) | 63 (1.3) | 46 (1.0) |  |
| 18.5-25.0 kg/m^2^ | 1020 (27.8) | 1070 (22.6) | 856 (18.8) |  |
| 25.0-30.0 kg/m^2^ | 1465 (39.9) | 1848 (39.0) | 1549 (34.0) |  |
| ≥30.0 kg/m^2^ | 1131 (30.8) | 1759 (37.1) | 2103 (46.2) |  |
|  |  |  |  |  |
| **Comorbidities and smoking** |  |  |  |  |
| Atrial fibrillation (history) | 1406 (38.3) | 1970 (41.6) | 2206 (48.4) | <0.001 |
| Hypertension | 2704 (73.6) | 3765 (79.4) | 3887 (85.2) | <0.001 |
| CHD‡ | 2028 (55.2) | 2804 (59.2) | 2911 (63.8) | <0.001 |
| Angina pectoris | 760 (20.7) | 1258 (26.5) | 1619 (35.5) | <0.001 |
| MI | 1240 (33.8) | 1679 (35.4) | 1716 (37.6) | <0.001 |
| Prior PCI/CABG | 1101 (30.0) | 1451 (30.6) | 1346 (29.5) | 0.60 |
| PCI | 757 (20.6) | 1021 (21.5) | 957 (21.0) | 0.73 |
| CABG | 501 (13.6) | 684 (14.4) | 654 (14.3) | 0.39 |
| Cerebral vascular disease |  |  |  |  |
| Stroke | 249 (6.8) | 414 (8.7) | 550 (12.1) | <0.001 |
| Prior TIA | 115 (3.1) | 194 (4.1) | 186 (4.1) | 0.033 |
| Non-cardiovascular systems |  |  |  |  |
| COPD/asthma | 472 (12.9) | 765 (16.1) | 959 (21.0) | <0.001 |
| Diabetes mellitus | 1220 (33.2) | 1770 (37.3) | 1875 (41.1) | <0.001 |
| Anaemia§ | 671 (18.6) | 1028 (22.1) | 1026 (23.1) | <0.001 |
| Current smoker | 445 (12.2) | 550 (11.6) | 521 (11.4) | 0.32 |
|  |  |  |  |  |
| **HF characteristics and investigations** |  |  |  |  |
| Time since HF diagnosis |  |  |  | <0.001 |
| $\leq$ 1 yr | 1397 (38.1) | 1630 (34.4) | 1370 (30.1) |  |
| > 1-5 yrs | 1318 (35.9) | 1731 (36.5) | 1788 (39.3) |  |
| > 5 yrs | 956 (26.0) | 1377 (29.1) | 1396 (30.7) |  |
| Previous hospitalization for HF | 2061 (56.1) | 2696 (56.9) | 2680 (58.8) | 0.014 |
| NYHA III/IV | 372 (10.1) | 904 (19.1) | 1709 (37.5) | <0.001 |
| KCCQ clinical summary score | 92 (84-98) | 80 (70-90) | 60 (47-73) | <0.001 |
| KCCQ total symptom score | 96 (87-100) | 83 (73-94) | 66 (51-79) | <0.001 |
| KCCQ overall summary score | 90 (82-96) | 78 (68-87) | 57 (45-70) | <0.001 |
| EQ-5D-3L VAS | 80 (70-90) | 70 (60-80) | 60 (50-70) | <0.001 |
| ECG findings and NT-proBNP |  |  |  |  |
| Atrial fibrillation/flutter | 925 (25.5) | 1263 (26.9) | 1408 (31.2) | <0.001 |
| Paced rhythm | 357 (9.8) | 504 (10.7) | 488 (10.8) | 0.17 |
| NT-proBNP, pg/ml | 1282 (724-2399) | 1300 (701-2566) | 1343 (688-2530) | 0.45 |
| Atrial fibrillation/flutter§§ | 1695 (1140-2661) | 1730 (1174-3005) | 1803 (1189-3166) | 0.002 |
| No atrial fibrillation/flutter§§ | 1104 (645-2228) | 1078 (605-2309) | 1047 (567-2167) | 0.003 |
| LVEF and other laboratory investigations |  |  |  |  |
| LVEF, % | 34 (28-49) | 35 (29-54) | 35 (30-55) | <0.001 |
| Haemoglobin, g/L | 140 (130-150) | 138 (127-149) | 136 (125-147) | <0.001 |
| Creatinine, μmol/L | 95 (80-112) | 95 (80-114) | 93 (78-112) | 0.013 |
| eGFR, mL/min/1.73m^2^ | 67 (54-80) | 64 (51-77) | 63 (50-76) | <0.001 |
| eGFR<60, mL/min/1.73m^2^ | 1326 (36.1) | 1993 (42.0) | 2004 (43.9) | <0.001 |
|  |  |  |  |  |
| **Medication and other interventions** |  |  |  |  |
| Diuretics | 3005 (81.8) | 4068 (85.8) | 4056 (88.9) | <0.001 |
| Loop | 2643 (72.0) | 3601 (76.0) | 3634 (79.7) | <0.001 |
| Thiazides | 342 (9.3) | 469 (9.9) | 427 (9.4) | 0.99 |
| Digitalis | 850 (23.1) | 1061 (22.4) | 1025 (22.5) | 0.49 |
| Beta-blocker | 3280 (89.3) | 4185 (88.3) | 3985 (87.4) | 0.007 |
| MRA | 1732 (47.2) | 2040 (43.0) | 2053 (45.0) | 0.08 |
| CCB | 556 (15.1) | 894 (18.9) | 979 (21.5) | <0.001 |
| Nitrates | 529 (14.4) | 797 (16.8) | 857 (18.8) | <0.001 |
| Statins | 2102 (57.2) | 2795 (59.0) | 2733 (59.9) | 0.015 |
| Antiarrhythmics | 389 (10.6) | 517 (10.9) | 471 (10.3) | 0.66 |
| Antiplatelet | 1613 (43.9) | 1884 (39.7) | 1787 (39.2) | <0.001 |
| Anticoagulant | 1080 (29.4) | 1511 (31.9) | 1591 (34.9) | <0.001 |
| Insulin of patients with diabetes | 283 (23.2) | 471 (26.6) | 595 (31.7) | <0.001 |
| Pacemaker | 371 (10.1) | 574 (12.1) | 575 (12.6) | <0.001 |
| ICD# | 381 (10.4) | 482 (10.2) | 380 (8.3) | 0.001 |
| CRT-P or CRT-D¶ | 162 (6.3) | 222 (7.5) | 179 (6.5) | 0.75 |

Data are presented as median (25%-75% quartile) for continuous measures, and n (%) for categorical measures.

† Including Central Europe and Russia.

‡CHD=angina, myocardial infarction, percutaneous coronary intervention, coronary artery bypass grafting, ischemic etiology.

§Hemoglobin<130g/L for male and 120g/L for female; §§ based on electrocardiogram.

# Including CRT-D.

¶ Only PARADIGM-HF.

BMI, body mass index; CABG, coronary artery bypass grafting; CCB, dihydropyridine calcium-channel blocker; CHD, coronary heart disease; COPD, chronic obstructive pulmonary disease; CRT-D, cardiac resynchronization therapy with defibrillator; CRT-P, cardiac resynchronization therapy with pacemaker; DBP, diastolic blood pressure; ECG, electrocardiogram; eGFR, estimated glomerular filtration rate; HF, heart failure; HR, heart rate; ICD, implantable cardioverter defibrillator; KCCQ, Kansas City Cardiomyopathy Questionnaire; LVEF, left ventricular ejection fraction; MAP, mean arterial pressure; MI, myocardial infarction; MRA, mineralocorticoid receptor antagonist; NT-proBNP, N-terminal pro B-type natriuretic peptide; NYHA, New York Heart Association; PCI, percutaneous coronary intervention; PP, pulse Pressure; SBP, systolic blood pressure; TIA, transient ischemic attack; VAS, Visual Analog Scale; yr, year.

Supplementary Table 6: Baseline characteristics according to baseline EuroQol 5-Dimension 3-Level (EQ-5D-3L) questionnaire Visual Analog Scale (VAS) category divided by tertile

|  | **Tertile 1: 0-59** | **Tertile 2: 60-79** | **Tertile 3: 80-100** | ***P* Value for trend** |
| --- | --- | --- | --- | --- |
| N, (%) | 3196 (24.7) | 5057 (39.1) | 4688 (36.2) |  |
| Age, yr | 68 (60-76) | 68 (61-76) | 67 (59-75) | <0.001 |
| Women | 1144 (35.8) | 1640 (32.4) | 1417 (30.2) |  |
| Race |  |  |  | <0.001 |
| White | 2598 (81.3) | 3827 (75.7) | 2853 (60.9) |  |
| Black | 110 (3.4) | 153 (3.0) | 265 (5.7) |  |
| Asian | 296 (9.3) | 766 (15.1) | 982 (20.9) |  |
| Others | 192 (6.0) | 311 (6.1) | 588 (12.5) |  |
| SBP, mmHg | 125 (112-135) | 125 (113-135) | 122 (110-135) | <0.001 |
| BMI, kg/m^2^ | 29.0 (25.5-33.0) | 28.5 (25.2-32.6) | 27.9 (24.8-31.6) | <0.001 |
| Atrial fibrillation (history) | 1588 (49.7) | 2256 (44.6) | 1717 (36.6) | <0.001 |
| MI | 1241 (38.8) | 1841 (36.4) | 1541 (32.9) | <0.001 |
| Stroke | 361 (11.3) | 481 (9.5) | 370 (7.9) | <0.001 |
| Diabetes mellitus | 1299 (40.6) | 1953 (38.6) | 1604 (34.2) | <0.001 |
| Time since HF diagnosis |  |  |  | <0.001 |
| $\leq$ 1 yr | 910 (28.5) | 1667 (33.0) | 1808 (38.6) |  |
| > 1-5 yrs | 1261 (39.5) | 1904 (37.7) | 1663 (35.5) |  |
| > 5 yrs | 1020 (32.0) | 1481 (29.3) | 1216 (25.9) |  |
| Previous hospitalization for HF | 1934 (60.5) | 2881 (57.0) | 2605 (55.6) | <0.001 |
| NYHA III/IV | 1220 (38.2) | 1224 (24.2) | 532 (11.4) | <0.001 |
| KCCQ clinical summary score | 62 (47-77) | 76 (63-87) | 89 (78-96) | <0.001 |
| NT-proBNP, pg/ml | 1448 (746-2889) | 1282 (699-2398) | 1245 (681-2365) | <0.001 |
| Atrial fibrillation/flutter§§ | 1965 (1215-3573) | 1722 (1153-2820) | 1688 (1134-2738) | <0.001 |
| No atrial fibrillation/flutter§§ | 1162 (628-2428) | 1042 (598-2165) | 1066 (601-2179) | 0.019 |
| LVEF, % | 34 (29-50) | 35 (30-54) | 35 (28-54) | 0.41 |
| Creatinine, μmol/L | 95 (80-114) | 94 (79-113) | 95 (80-112) | 0.47 |
| eGFR, mL/min/1.73m^2^ | 63 (50-77) | 64 (51-77) | 65 (53-78) | <0.001 |
| Diuretics | 2829 (88.5) | 4368 (86.4) | 3902 (83.2) | <0.001 |
| Beta-blocker | 2833 (88.6) | 4462 (88.2) | 4124 (88.0) | 0.37 |
| MRA | 1481 (46.3) | 2172 (43.0) | 2162 (46.1) | 0.81 |
| Pacemaker | 438 (13.7) | 619 (12.2) | 459 (9.8) | <0.001 |
| ICD# | 353 (11.0) | 491 (9.7) | 397 (8.5) | <0.001 |

Definitions and abbreviations as in **Supplementary Table 4**.

Supplementary Table 7: Patients completed the instrument in the cohort of PARADIGM-HF (HFrEF) and PARAGON-HF (HFpEF) at baseline.

| EQ-5D-3L | | HFrEF | HFpEF |
| --- | --- | --- | --- |
| Mobility | No problem | 4689 (56.6) | 2178 (46.3) |
|  | Some problem | 3571 (43.1) | 2521 (53.6) |
|  | Extreme problem | 19 (0.2) | 8 (0.2) |
| Self-care | No problem | 6929 (83.7) | 3933 (83.6) |
|  | Some problem | 1317 (15.9) | 733 (15.6) |
|  | Extreme problem | 32 (0.4) | 41 (0.9) |
| Usual activities | No problem | 4835 (58.4) | 2598 (55.2) |
|  | Some problem | 3238 (39.1) | 1995 (42.4) |
|  | Extreme problem | 204 (2.5) | 114 (2.4) |
| Pain/discomfort | No problem | 4645 (56.1) | 2221 (47.2) |
|  | Some problem | 3471 (41.9) | 2279 (48.4) |
|  | Extreme problem | 160 (1.9) | 205 (4.4) |
| Anxiety/depression | No problem | 5591 (67.5) | 3183 (67.7) |
|  | Some problem | 2534 (30.6) | 1430 (30.4) |
|  | Extreme problem | 152 (1.8) | 92 (2.0) |

Data are presented as n (%).

EQ-5D-3L, EuroQol 5-Dimension 3-Level questionnaire. HFrEF, heart failure with reduced ejection fraction; HFpEF, heart failure with preserved ejection fraction; PARADIGM-HF, Prospective Comparison of ARNI with ACEI to Determine Impact on Global Mortality and Morbidity in Heart Failure; PARAGON-HF, Prospective Comparison of ARNI with ARB Global Outcomes in HF With Preserved Ejection Fraction.

The total number of patients completed each dimension of the instrument in PARADIGM-HF (HFrEF) and PARAGON-HF (HFpEF) can be found in **Supplementary Table 2** and **Supplementary Table 3**, respectively.

Supplementary Table 8: Baseline characteristics based on answers to the EuroQol 5-Dimension 3-Level (EQ-5D-3L) “mobility” question.

|  | **No problem** | **Some problem** | **Extreme problem** |
| --- | --- | --- | --- |
| N, (%) | 6867 (52.9) | 6092 (46.9) | 27 (0.2) |
| Age, yr | 66 (58-74) | 70 (62-77) | 71 (66-78) |
| Women | 1849 (26.9) | 2356 (38.7) | 12 (44.4) |
| Race |  |  |  |
| White | 4373 (63.7) | 4936 (81.0) | 13 (48.1) |
| Black | 330 (4.8) | 196 (3.2) | 3 (11.1) |
| Asian | 1452 (21.1) | 585 (9.6) | 7 (25.9) |
| Others | 712 (10.4) | 375 (6.2) | 4 (14.8) |
| SBP, mmHg | 121 (110-134) | 125 (114-137) | 123 (120-137) |
| BMI, kg/m^2^ | 27.6 (24.5-31.1) | 29.4 (25.9-33.5) | 27.8 (23.6-32.6) |
| Atrial fibrillation (history) | 2610 (38.0) | 2963 (48.7) | 13 (48.1) |
| MI | 2343 (34.1) | 2284 (37.5) | 12 (44.4) |
| Stroke | 517 (7.5) | 690 (11.3) | 7 (25.9) |
| Diabetes Mellitus | 2351 (34.2) | 2504 (41.1) | 13 (48.1) |
| Time since HF diagnosis |  |  |  |
| $\leq$ 1 yr | 2556 (37.3) | 1835 (30.1) | 7 (25.9) |
| > 1-5 yrs | 2464 (35.9) | 2370 (38.9) | 10 (37.0) |
| > 5 yrs | 1841 (26.8) | 1882 (30.9) | 10 (37.0) |
| Previous hospitalization for HF | 3903 (56.8) | 3521 (57.8) | 20 (74.1) |
| NYHA III/IV | 929 (13.5) | 2044 (33.6) | 12 (44.4) |
| KCCQ clinical summary score | 88 (77-96) | 66 (51-78) | 50 (37-81) |
| NT-proBNP, pg/ml | 1287 (707-2441) | 1332 (699-2592) | 1158 (547-2983) |
| Atrial fibrillation/flutter§§ | 1729 (1168-2848) | 1787 (1172-3150) | 1622 (962-4387) |
| No atrial fibrillation/flutter§§ | 1089 (627-2242) | 1053 (572-2230) | 1010 (485-2186) |
| LVEF, % | 34 (28-50) | 35 (30-55) | 34 (30-60) |
| Creatinine, μmol/L | 94 (80-112) | 95 (80-115) | 100 (90-111) |
| eGFR, mL/min/1.73m^2^ | 66 (53-79) | 62 (50-76) | 56 (45-70) |
| Diuretics | 5703 (83.0) | 5411 (88.8) | 26 (96.3) |
| Beta-blocker | 6124 (89.2) | 5311 (87.2) | 24 (88.9) |
| MRA | 3149 (45.9) | 2667 (43.8) | 14 (51.9) |
| Pacemaker | 762 (11.1) | 758 (12.4) | 3 (11.1) |
| ICD# | 723 (10.5) | 520 (8.5) | 2 (7.4) |

Definitions and abbreviations as in **Supplementary Table 4**.

Supplementary Table 9: Baseline characteristics based on answers to the EuroQol 5-Dimension 3-Level (EQ-5D-3L) “self-care” question.

|  | **No problem** | **Some problem** | **Extreme problem** |
| --- | --- | --- | --- |
| N, (%) | 10862 (83.7) | 2050 (15.8) | 73 (0.6) |
| Age, yr | 68 (60-75) | 70 (62-77) | 77 (67-82) |
| Women | 3360 (30.9) | 817 (39.9) | 40 (54.8) |
| Race |  |  |  |
| White | 7620 (70.2) | 1650 (80.5) | 51 (69.9) |
| Black | 464 (4.3) | 61 (3.0) | 4 (5.5) |
| Asian | 1825 (16.8) | 205 (10.0) | 14 (19.2) |
| Others | 953 (8.8) | 134 (6.5) | 4 (5.5) |
| SBP, mmHg | 123 (110-135) | 125 (115-136) | 126 (115-139) |
| BMI, kg/m^2^ | 28.1 (25.0-32.0) | 29.5 (25.8-34.1) | 29.3 (25.9-33.8) |
| Atrial fibrillation (history) | 8549 (78.7) | 1747 (85.2) | 68 (93.2) |
| MI | 3327 (30.6) | 559 (27.3) | 17 (23.3) |
| Stroke | 881 (8.1) | 315 (15.4) | 18 (24.7) |
| Diabetes Mellitus | 3951 (36.4) | 877 (42.8) | 39 (53.4) |
| Time since HF diagnosis |  |  |  |
| $\leq$ 1 yr | 3796 (35.0) | 582 (28.4) | 21 (28.8) |
| > 1-5 yrs | 4013 (37.0) | 799 (39.0) | 31 (42.5) |
| > 5 yrs | 3045 (28.1) | 666 (32.5) | 21 (28.8) |
| Previous hospitalization for HF | 6134 (56.5) | 1267 (61.8) | 42 (57.5) |
| NYHA III/IV | 2051 (18.9) | 904 (44.2) | 30 (41.1) |
| KCCQ clinical summary score | 82 (68-92) | 54 (42-67) | 43 (30-65) |
| NT-proBNP, pg/ml | 1282 (697-2441) | 1462 (750-2970) | 1267 (642-2124) |
| Atrial fibrillation/flutter§§ | 1735 (1162-2911) | 1853 (1189-3425) | 2102 (1445-2983) |
| No atrial fibrillation/flutter§§ | 1060 (605-2191) | 1183 (608-2564) | 923 (588-1582) |
| LVEF, % | 35 (29-53) | 35 (30-53) | 49 (33-60) |
| Creatinine, μmol/L | 95 (80-113) | 94 (78-114) | 93 (74-111) |
| eGFR, mL/min/1.73m^2^ | 64 (52-78) | 63 (50-76) | 62 (45-73) |
| Diuretics | 6.2 (5.8-6.8) | 6.3 (5.8-7.1) | 6.2 (5.8-7.9) |
| Beta-blocker | 2439 (22.5) | 481 (23.5) | 19 (26.0) |
| MRA | 9596 (88.3) | 1808 (88.2) | 55 (75.3) |
| Pacemaker | 1272 (11.7) | 245 (12.0) | 6 (8.2) |
| ICD# | 1089 (10.0) | 150 (7.3) | 6 (8.2) |

Definitions and abbreviations as in **Supplementary Table 4**.

Supplementary Table 10: Baseline characteristics based on answers to the EuroQol 5-Dimension 3-Level (EQ-5D-3L) “usual activities” question.

|  | **No problem** | **Some problem** | **Extreme problem** |
| --- | --- | --- | --- |
| N, (%) | 7433 (57.2) | 5233 (40.3) | 318 (2.4) |
| Age, yr | 68 (60-75) | 68 (60-76) | 69 (61-78) |
| Women | 2035 (27.4) | 2053 (39.2) | 129 (40.6) |
| Race |  |  |  |
| White | 4937 (66.4) | 4146 (79.2) | 237 (74.5) |
| Black | 314 (4.2) | 197 (3.8) | 18 (5.7) |
| Asian | 1464 (19.7) | 542 (10.4) | 38 (11.9) |
| Others | 718 (9.7) | 348 (6.7) | 25 (7.9) |
| SBP, mmHg | 122 (110-135) | 125 (113-135) | 123 (110-136) |
| BMI, kg/m^2^ | 27.9 (24.8-31.6) | 29.1 (25.6-33.2) | 29.6 (25.4-34.5) |
| Atrial fibrillation (history) | 2962 (39.9) | 2478 (47.4) | 146 (45.9) |
| MI | 2568 (34.5) | 1944 (37.1) | 126 (39.6) |
| Stroke | 584 (7.9) | 576 (11.0) | 54 (17.0) |
| Diabetes Mellitus | 2653 (35.7) | 2065 (39.5) | 149 (46.9) |
| Time since HF diagnosis |  |  |  |
| $\leq$ 1 yr | 2695 (36.3) | 1622 (31.0) | 82 (25.8) |
| > 1-5 yrs | 2702 (36.4) | 2008 (38.4) | 133 (41.8) |
| > 5 yrs | 2033 (27.4) | 1595 (30.5) | 103 (32.4) |
| Previous hospitalization for HF | 4135 (55.6) | 3095 (59.1) | 213 (67.0) |
| NYHA III/IV | 1049 (14.1) | 1779 (34.0) | 157 (49.4) |
| KCCQ clinical summary score | 88 (77-95) | 65 (51-77) | 43 (28-55) |
| NT-proBNP, pg/ml | 1272 (702-2424) | 1361 (707-2609) | 1370 (654-2876) |
| Atrial fibrillation/flutter§§ | 1695 (1153-2800) | 1834 (1197-3226) | 1931 (1096-2983) |
| No atrial fibrillation/flutter§§ | 1072 (617-2230) | 1078 (589-2228) | 1111 (520-2686) |
| LVEF, % | 35 (29-51) | 35 (30-55) | 35 (28-52) |
| Creatinine, μmol/L | 95 (80-113) | 94 (79-113) | 98 (82-115) |
| eGFR, mL/min/1.73m^2^ | 65 (53-79) | 63 (50-76) | 61 (48-74) |
| Diuretics | 6214 (83.6) | 4633 (88.5) | 291 (91.5) |
| Beta-blocker | 6567 (88.3) | 4621 (88.3) | 271 (85.2) |
| MRA | 3289 (44.2) | 2376 (45.4) | 165 (51.9) |
| Pacemaker | 801 (10.8) | 678 (13.0) | 44 (13.8) |
| ICD# | 693 (9.3) | 512 (9.8) | 40 (12.6) |

Definitions and abbreviations as in **Supplementary Table 4**.

Supplementary Table 11: Baseline characteristics based on answers to the EuroQol 5-Dimension 3-Level (EQ-5D-3L) “pain/discomfort” question.

|  | **No problem** | **Some problem** | **Extreme problem** |
| --- | --- | --- | --- |
| N, (%) | 6866 (52.9) | 5750 (44.3) | 365 (2.8) |
| Age, yr | 67 (59-74) | 69 (61-76) | 73 (65-78) |
| Women | 1833 (26.7) | 2182 (37.9) | 201 (55.1) |
| Race |  |  |  |
| White | 4643 (67.6) | 4381 (76.2) | 295 (80.8) |
| Black | 286 (4.2) | 218 (3.8) | 24 (6.6) |
| Asian | 1296 (18.9) | 732 (12.7) | 15 (4.1) |
| Others | 641 (9.3) | 419 (7.3) | 31 (8.5) |
| SBP, mmHg | 122 (110-134) | 125 (113-136) | 125 (112-140) |
| BMI, kg/m^2^ | 27.8 (24.7-31.6) | 29.0 (25.5-32.9) | 30.7 (27.2-35.1) |
| Atrial fibrillation (history) | 2793 (40.7) | 2609 (45.4) | 183 (50.1) |
| MI | 2420 (35.2) | 2105 (36.6) | 112 (30.7) |
| Stroke | 574 (8.4) | 585 (10.2) | 54 (14.8) |
| Diabetes Mellitus | 2434 (35.5) | 2272 (39.5) | 161 (44.1) |
| Time since HF diagnosis |  |  |  |
| $\leq$ 1 yr | 2484 (36.2) | 1806 (31.4) | 109 (29.9) |
| > 1-5 yrs | 2492 (36.3) | 2210 (38.5) | 138 (37.9) |
| > 5 yrs | 1887 (27.5) | 1727 (30.1) | 117 (32.1) |
| Previous hospitalization for HF | 3938 (57.4) | 3288 (57.2) | 215 (58.9) |
| NYHA III/IV | 1120 (16.3) | 1729 (30.1) | 136 (37.3) |
| KCCQ clinical summary score | 86 (74-95) | 68 (54-82) | 52 (37-70) |
| NT-proBNP, pg/ml | 1315 (728-2541) | 1292 (679-2477) | 1269 (699-2258) |
| Atrial fibrillation/flutter§§ | 1752 (1155-2915) | 1778 (1181-3067) | 1735 (1162-2924) |
| No atrial fibrillation/flutter§§ | 1126 (635-2326) | 1010 (572-2154) | 1002 (520-2042) |
| LVEF, % | 34 (28-50) | 35 (30-55) | 48 (32-60) |
| Creatinine, μmol/L | 95 (80-113) | 94 (79-113) | 95 (80-118) |
| eGFR, mL/min/1.73m^2^ | 65 (53-78) | 63 (51-77) | 58 (46-72) |
| Diuretics | 5770 (84.0) | 5024 (87.4) | 341 (93.4) |
| Beta-blocker | 6105 (88.9) | 5049 (87.8) | 301 (82.5) |
| MRA | 3175 (46.2) | 2516 (43.8) | 136 (37.3) |
| Pacemaker | 763 (11.1) | 705 (12.3) | 52 (14.2) |
| ICD# | 737 (10.7) | 469 (8.2) | 38 (10.4) |

Definitions and abbreviations as in **Supplementary Table 4**.

Supplementary Table 12: Baseline characteristics based on answers to the EuroQol 5-Dimension 3-Level (EQ-5D-3L) “anxiety/depression” question.

|  | **No problem** | **Some problem** | **Extreme problem** |
| --- | --- | --- | --- |
| N, (%) | 8744 (67.6) | 3964 (30.5) | 244 (1.9) |
| Age, yr | 68 (60-76) | 67 (59-75) | 68 (59-75) |
| Women | 2471 (28.2) | 1619 (40.8) | 126 (51.6) |
| Race |  |  |  |
| White | 6150 (70.1) | 2992 (75.5) | 176 (72.1) |
| Black | 376 (4.3) | 141 (3.6) | 12 (4.9) |
| Asian | 1508 (17.2) | 507 (12.8) | 29 (11.9) |
| Others | 740 (8.4) | 324 (8.2) | 27 (11.1) |
| SBP, mmHg | 123 (111-135) | 124 (113-135) | 128 (115-140) |
| BMI, kg/m^2^ | 28.3 (25.0-32.1) | 28.5 (25.3-32.7) | 29.7 (26.7-34.3) |
| Atrial fibrillation (history) | 3726 (42.5) | 1751 (44.2) | 109 (44.7) |
| MI | 3094 (35.3) | 1472 (37.1) | 72 (29.5) |
| Stroke | 777 (8.9) | 407 (10.3) | 30 (12.3) |
| Diabetes Mellitus | 3246 (37.0) | 1524 (38.4) | 98 (40.2) |
| Time since HF diagnosis |  |  |  |
| $\leq$ 1 yr | 3022 (34.5) | 1296 (32.7) | 80 (32.8) |
| > 1-5 yrs | 3233 (36.9) | 1505 (38.0) | 102 (41.8) |
| > 5 yrs | 2514 (28.7) | 1157 (29.2) | 62 (25.4) |
| Previous hospitalization for HF | 4953 (56.5) | 2336 (58.9) | 152 (62.3) |
| NYHA III/IV | 1683 (19.2) | 1208 (30.5) | 94 (38.5) |
| KCCQ clinical summary score | 82 (69-93) | 67 (52-82) | 52 (36-69) |
| NT-proBNP, pg/ml | 1303 (712-2524) | 1319 (690-2484) | 1326 (648-2240) |
| Atrial fibrillation/flutter§§ | 1754 (1173-2943) | 1773 (1151-3021) | 1775 (1243-3943) |
| No atrial fibrillation/flutter§§ | 1072 (614-2277) | 1088 (586-2199) | 1022 (481-2070) |
| LVEF, % | 35 (29-53) | 35 (30-54) | 35 (30-55) |
| Creatinine, μmol/L | 95 (80-114) | 92 (77-110) | 93 (74-110) |
| eGFR, mL/min/1.73m^2^ | 64 (51-77) | 64 (52-78) | 64 (51-75) |
| Diuretics | 7472 (85.2) | 3449 (87.0) | 216 (88.5) |
| Beta-blocker | 7739 (88.2) | 3507 (88.5) | 211 (86.5) |
| MRA | 3909 (44.6) | 1817 (45.8) | 102 (41.8) |
| Pacemaker | 1060 (12.1) | 440 (11.1) | 23 (9.4) |
| ICD# | 894 (10.2) | 336 (8.5) | 14 (5.7) |

Definitions and abbreviations as in **Supplementary Table 4**.

Supplementary Table 13: Effect of sacubitril/valsartan versus active comparator on EuroQol 5-Dimension 3-Level (EQ-5D-3L) questionnaire Visual Analog Scale (VAS) over time (8 months)

|  | **Mean ± SD** | | **Median (IQR)** | | **48 weeks from baseline** | |
| --- | --- | --- | --- | --- | --- | --- |
|  | **Baseline** | **8 months** | **Baseline** | **8 months** | **Mean change** | **Mean difference (95% CI)** |
| Sacubitril/valsartan (n=5927) | 68.4±18.7 | 70.4±17.8 | 70.0 (60.0-80.0) | 70.0 (60.0-80.0) | 1.71 (1.32 to 2.10) | 0.53 (-0.02 to 1.09)  P=0.059 |
| Active comparator  (n=5838) | 68.1±18.9 | 69.8±18.1 | 70.0 (59.0-80.0) | 70.0 (60.0-80.0) | 1.18 (0.78 to 1.77) |  |

*Patients were included in this analysis if they had both available VAS at baseline and 48 weeks follow-up. Analysis of covariance (ANCOVA) adjusted for baseline value was performed to test for the treatment effect of o sacubitril/valsartan versus active comparator.

Supplementary Table 14: Clinical outcomes according to baseline EuroQol 5-Dimension 3-Level (EQ-5D-3L) Level Sum Score (LSS) category divided by tertile.

|  | **Tertile 1: 5** | **Tertile 2: 6-7** | **Tertile 3: 8-15** |
| --- | --- | --- | --- |
| N, (%) | 3673 (28.3) | 4740 (36.5) | 4561 (35.2) |
| **CV death or HF hospitalization** |  |  |  |
| Number of events (%) | 725 (19.7) | 1089 (23.0) | 1236 (27.1) |
| Rate per 100 patient-years (95% CI) | 8.6 (8.0-9.3) | 10.0 (9.4-10.6) | 12.2 (11.6-12.9) |
| Unadjusted HR (95% CI)^a^ | 1.00 (Ref.) | 1.21 (1.10-1.33) | 1.50 (1.37-1.65) |
| Additional adjusted HR (95% CI)^b^ | 1.00 (Ref.) | 1.13 (1.03-1.24) | 1.32 (1.19-1.45) |
| **First HF hospitalization** |  |  |  |
| Number of events (%) | 458 (12.5) | 740 (15.6) | 788 (17.3) |
| Rate per 100 patient-years (95% CI) | 5.5 (5.0-6.0) | 6.8 (6.3-7.3) | 7.8 (7.3-8.4) |
| Unadjusted HR (95% CI)^a^ | 1.00 (Ref.) | 1.27 (1.13-1.43) | 1.46 (1.30-1.64) |
| Additional adjusted HR (95% CI)^b^ | 1.00 (Ref.) | 1.17 (1.04-1.32) | 1.27 (1.12-1.43) |
| **CV death** |  |  |  |
| Number of events (%) | 388 (10.6) | 562 (11.9) | 692 (15.2) |
| Rate per 100 patient-years (95% CI) | 4.3 (3.9-4.8) | 4.8 (4.4-5.2) | 6.2 (5.8-6.7) |
| Unadjusted HR (95% CI)^a^ | 1.00 (Ref.) | 1.20 (1.06-1.37) | 1.65 (1.45-1.87) |
| Additional adjusted HR (95% CI)^b^ | 1.00 (Ref.) | 1.11 (0.98-1.27) | 1.44 (1.26-1.64) |
| **Non-CV death** |  |  |  |
| Number of events (%) | 109 (3.0) | 147 (3.1) | 185 (4.1) |
| Rate per 100 patient-years (95% CI) | 1.2 (1.0-1.5) | 1.2 (1.1-1.5) | 1.7 (1.4-1.9) |
| Unadjusted HR (95% CI)^a^ | 1.00 (Ref.) | 0.99 (0.77-1.27) | 1.33 (1.05-1.70) |
| Additional adjusted HR (95% CI)^b^ | 1.00 (Ref.) | 0.96 (0.75-1.23) | 1.24 (0.96-1.60) |
| **All-cause death** |  |  |  |
| Number of events (%) | 527 (14.4) | 751 (15.8) | 925 (20.3) |
| Rate per 100 patient-years (95% CI) | 5.9 (5.4-6.4) | 6.4 (5.9-6.8) | 8.3 (7.8-8.9) |
| Unadjusted HR (95% CI)^a^ | 1.00 (Ref.) | 1.15 (1.03-1.29) | 1.56 (1.40-1.74) |
| Additional adjusted HR (95% CI)^b^ | 1.00 (Ref.) | 1.07 (0.96-1.20) | 1.39 (1.24-1.56) |
| **Total HF hospitalizations/CV death** |  |  |  |
| Number of events | 1121 | 1829 | 2031 |
| Rate per 100 patient-years (95% CI) | 12.5 (11.5-13.7) | 15.5 (14.4-16.7) | 18.3 (17.1-19.6) |
| Unadjusted RR (95% CI)^c^ | 1.00 (Ref.) | 1.32 (1.17-1.49) | 1.66 (1.47-1.88) |
| Adjusted RR (95% CI)^b^ | 1.00 (Ref.) | 1.18 (1.04-1.32) | 1.40 (1.23-1.58) |

Definitions and abbreviations as in **Table 3**.

Supplementary Table 15: Clinical outcomes according to baseline EuroQol 5-Dimension 3-Level (EQ-5D-3L) Level Sum Score (LSS) category divided by tertile in HFrEF

|  | **Tertile 1: 5** | **Tertile 2: 6-7** | **Tertile 3: 8-15** |
| --- | --- | --- | --- |
| N, (%) | 2582 (31.2) | 2944 (35.6) | 2745 (33.2) |
| **CV death or HF hospitalization** |  |  |  |
| Number of events (%) | 535 (20.7) | 684 (23.2) | 779 (28.4) |
| Rate per 100 patient-years (95% CI) | 9.8 (9-10.7) | 11.2 (10.4-12.1) | 14.4 (13.4-15.4) |
| Unadjusted HR (95% CI)^a^ | 1.00 (Ref.) | 1.14 (1.02-1.28) | 1.47 (1.31-1.64) |
| Additional adjusted HR (95% CI)^b^ | 1.00 (Ref.) | 1.07 (0.95-1.20) | 1.26 (1.12-1.42) |
| **First HF hospitalization** |  |  |  |
| Number of events (%) | 303 (11.7) | 429 (14.6) | 444 (16.2) |
| Rate per 100 patient-years (95% CI) | 5.6 (5-6.2) | 7 (6.4-7.7) | 8.2 (7.5-9) |
| Unadjusted HR (95% CI)^a^ | 1.00 (Ref.) | 1.26 (1.08-1.45) | 1.44 (1.24-1.67) |
| Additional adjusted HR (95% CI)^b^ | 1.00 (Ref.) | 1.16 (1.00-1.35) | 1.23 (1.05-1.44) |
| **CV death** |  |  |  |
| Number of events (%) | 331 (12.8) | 397 (13.5) | 504 (18.4) |
| Rate per 100 patient-years (95% CI) | 5.7 (5.1-6.4) | 6.1 (5.5-6.7) | 8.5 (7.8-9.3) |
| Unadjusted HR (95% CI)^a^ | 1.00 (Ref.) | 1.08 (0.93-1.25) | 1.57 (1.36-1.81) |
| Additional adjusted HR (95% CI)^b^ | 1.00 (Ref.) | 1.00 (0.86-1.16) | 1.34 (1.15-1.55) |
| **Non-CV death** |  |  |  |
| Number of events (%) | 55 (2.1) | 93 (3.2) | 78 (2.8) |
| Rate per 100 patient-years (95% CI) | 0.9 (0.7-1.2) | 1.4 (1.2-1.7) | 1.3 (1.1-1.6) |
| Unadjusted HR (95% CI)^a^ | 1.00 (Ref.) | 1.48 (1.06-2.07) | 1.39 (0.98-1.98) |
| Additional adjusted HR (95% CI)^b^ | 1.00 (Ref.) | 1.46 (1.04-2.05) | 1.37 (0.95-1.98) |
| **All-cause death** |  |  |  |
| Number of events (%) | 408 (15.8) | 508 (17.3) | 608 (22.2) |
| Rate per 100 patient-years (95% CI) | 7.0 (6.4-7.8) | 7.7 (7.1-8.4) | 10.3 (9.5-11.1) |
| Unadjusted HR (95% CI)^a^ | 1.00 (Ref.) | 1.11 (0.98-1.27) | 1.52 (1.34-1.73) |
| Additional adjusted HR (95% CI)^b^ | 1.00 (Ref.) | 1.04 (0.91-1.19) | 1.33 (1.16-1.52) |
| **Total HF hospitalizations/CV death** |  |  |  |
| Number of events | 793 | 1120 | 1222 |
| Rate per 100 patient-years (95% CI) | 13.7 (12.4-15.1) | 17.1 (15.6-18.7) | 20.7 (19.0-22.5) |
| Unadjusted RR (95% CI)^c^ | 1.00 (Ref.) | 1.27 (1.09-1.48) | 1.66 (1.43-1.93) |
| Adjusted RR (95% CI)^b^ | 1.00 (Ref.) | 1.13 (0.97-1.30) | 1.30 (1.12-1.51) |

Definitions and abbreviations as in **Table 3**.

Supplementary Table 16: Clinical outcomes according to baseline EuroQol 5-Dimension 3-Level (EQ-5D-3L) Level Sum Score (LSS) category divided by tertile in HFmrEF/HFpEF

|  | **Tertile 1: 5** | **Tertile 2: 6-7** | **Tertile 3: 8-15** |
| --- | --- | --- | --- |
| N, (%) | 1091 (23.2) | 1796 (38.2) | 1816 (38.6) |
| **CV death or HF hospitalization** |  |  |  |
| Number of events (%) | 190 (17.2) | 405 (22.6) | 457 (25.2) |
| Rate per 100 patient-years (95% CI) | 6.5 (5.6-7.5) | 8.4 (7.7-9.3) | 9.8 (8.9-10.7) |
| Unadjusted HR (95% CI)^a^ | 1.00 (Ref.) | 1.38 (1.16-1.64) | 1.61 (1.36-1.92) |
| Additional adjusted HR (95% CI)^b^ | 1.00 (Ref.) | 1.26 (1.06-1.51) | 1.43 (1.19-1.71) |
| **First HF hospitalization** |  |  |  |
| Number of events (%) | 155 (14.2) | 311 (17.3) | 344 (18.9) |
| Rate per 100 patient-years (95% CI) | 5.3 (4.5-6.2) | 6.5 (5.8-7.2) | 7.3 (6.6-8.2) |
| Unadjusted HR (95% CI)^a^ | 1.00 (Ref.) | 1.30 (1.07-1.57) | 1.49 (1.23-1.81) |
| Additional adjusted HR (95% CI)^b^ | 1.00 (Ref.) | 1.18 (0.97-1.43) | 1.3 (1.06-1.59) |
| **CV death** |  |  |  |
| Number of events (%) | 57 (5.2) | 165 (9.2) | 188 (10.4) |
| Rate per 100 patient-years (95% CI) | 1.8 (1.4-2.3) | 3.1 (2.7-3.7) | 3.6 (3.1-4.2) |
| Unadjusted HR (95% CI)^a^ | 1.00 (Ref.) | 1.85 (1.36-2.5) | 2.13 (1.58-2.87) |
| Additional adjusted HR (95% CI)^b^ | 1.00 (Ref.) | 1.67 (1.23-2.27) | 1.97 (1.44-2.7) |
| **Non-CV death** |  |  |  |
| Number of events (%) | 54 (5.0) | 54 (3.0) | 107 (5.9) |
| Rate per 100 patient-years (95% CI) | 1.7 (1.3-2.2) | 1 (0.8-1.3) | 2.1 (1.7-2.5) |
| Unadjusted HR (95% CI)^a^ | 1.00 (Ref.) | 0.59 (0.4-0.86) | 1.20 (0.86-1.66) |
| Additional adjusted HR (95% CI)^b^ | 1.00 (Ref.) | 0.56 (0.38-0.82) | 1.05 (0.73-1.5) |
| **All-cause death** |  |  |  |
| Number of events (%) | 119 (10.9) | 243 (13.5) | 317 (17.5) |
| Rate per 100 patient-years (95% CI) | 3.8 (3.1-4.5) | 4.6 (4.1-5.3) | 6.1 (5.5-6.8) |
| Unadjusted HR (95% CI)^a^ | 1.00 (Ref.) | 1.26 (1.01-1.57) | 1.67 (1.35-2.07) |
| Additional adjusted HR (95% CI)^b^ | 1.00 (Ref.) | 1.16 (0.93-1.46) | 1.55 (1.24-1.94) |
| **Total HF hospitalizations/CV death** |  |  |  |
| Number of events | 328 | 709 | 809 |
| Rate per 100 patient-years (95% CI) | 10.4 (8.7-12.3) | 13.5 (12.0-15.3) | 15.6 (13.9-17.4) |
| Unadjusted RR (95% CI)^c^ | 1.00 (Ref.) | 1.42 (1.14-1.75) | 1.67 (1.35-2.07) |
| Adjusted RR (95% CI)^b^ | 1.00 (Ref.) | 1.28 (1.04-1.58) | 1.58 (1.27-1.97) |

Definitions and abbreviations as in **Table 4**.

Supplementary Table 17: Clinical outcomes according to baseline EuroQol 5-Dimension 3-Level (EQ-5D-3L) questionnaire Visual Analog Scale (VAS) category divided by tertile.

|  | **Tertile 1: 0-59** | **Tertile 2: 60-79** | **Tertile 3: 80-100** |
| --- | --- | --- | --- |
| N, (%) | 3196 (24.7) | 5057 (39.1) | 4688 (36.2) |
| **CV death or HF hospitalization** |  |  |  |
| Number of events (%) | 882 (27.6) | 1223 (24.2) | 942 (20.1) |
| Rate per 100 patient-years (95% CI) | 12.6 (11.8-13.5) | 10.6 (10.0-11.2) | 8.7 (8.2-9.3) |
| Unadjusted HR (95% CI)^a^ | 1.00 (Ref.) | 0.84 (0.77-0.92) | 0.67 (0.61-0.74) |
| Additional adjusted HR (95% CI)^b^ | 1.00 (Ref.) | 0.91 (0.83-0.99) | 0.79 (0.71-0.87) |
| **First HF hospitalization** |  |  |  |
| Number of events (%) | 560 (17.5) | 822 (16.3) | 604 (12.9) |
| Rate per 100 patient-years (95% CI) | 8.0 (7.4-8.7) | 7.1 (6.7-7.6) | 5.6 (5.2-6.1) |
| Unadjusted HR (95% CI)^a^ | 1.00 (Ref.) | 0.88 (0.79-0.99) | 0.68 (0.61-0.77) |
| Additional adjusted HR (95% CI)^b^ | 1.00 (Ref.) | 0.95 (0.85-1.06) | 0.80 (0.71-0.91) |
| **CV death** |  |  |  |
| Number of events (%) | 502 (15.7) | 637 (12.6) | 498 (10.6) |
| Rate per 100 patient-years (95% CI) | 6.5 (6.0-7.1) | 5.1 (4.7-5.5) | 4.3 (4.0-4.7) |
| Unadjusted HR (95% CI)^a^ | 1.00 (Ref.) | 0.79 (0.70-0.89) | 0.63 (0.55-0.71) |
| Additional adjusted HR (95% CI)^b^ | 1.00 (Ref.) | 0.85 (0.76-0.96) | 0.72 (0.63-0.83) |
| **Non-CV death** |  |  |  |
| Number of events (%) | 144 (4.5) | 171 (3.4) | 121 (2.6) |
| Rate per 100 patient-years (95% CI) | 1.9 (1.6-2.2) | 1.4 (1.2-1.6) | 1.0 (0.9-1.3) |
| Unadjusted HR (95% CI)^a^ | 1.00 (Ref.) | 0.72 (0.58-0.90) | 0.54 (0.42-0.70) |
| Additional adjusted HR (95% CI)^b^ | 1.00 (Ref.) | 0.75 (0.60-0.94) | 0.60 (0.46-0.78) |
| **All-cause death** |  |  |  |
| Number of events (%) | 680 (21.3) | 856 (16.9) | 656 (14.0) |
| Rate per 100 patient-years (95% CI) | 8.8 (8.2-9.5) | 6.8 (6.4-7.3) | 5.7 (5.3-6.1) |
| Unadjusted HR (95% CI)^a^ | 1.00 (Ref.) | 0.78 (0.71-0.86) | 0.61 (0.55-0.68) |
| Additional adjusted HR (95% CI)^b^ | 1.00 (Ref.) | 0.83 (0.75-0.92) | 0.70 (0.62-0.78) |
| **Total HF hospitalizations/CV death** |  |  |  |
| Number of events | 1450 | 1979 | 1547 |
| Rate per 100 patient-years (95% CI) | 18.8 (17.3-20.4) | 15.8 (14.8-16.9) | 13.4 (12.4-14.5) |
| Unadjusted RR (95% CI)^c^ | 1.00 (Ref.) | 0.83 (0.73-0.94) | 0.64 (0.56-0.73) |
| Adjusted RR (95% CI)^b^ | 1.00 (Ref.) | 0.91 (0.81-1.03) | 0.78 (0.69-0.88) |

Definitions and abbreviations as in **Table 3**.

Supplementary Table 18: Association of 8-month EuroQol 5-Dimension 3-Level (EQ-5D-3L) questionnaire Level Sum Score (LSS) with clinical outcomes

|  | **LSS: 5** | **LSS: 6-7** | **LSS: 8-15** |
| --- | --- | --- | --- |
| N, (%) | 3582 (30.5) | 3981 (34.0) | 4164 (35.5) |
| **CV death or HF hospitalization** |  |  |  |
| Number of events (%) | 479 (13.4) | 709 (17.8) | 909 (21.8) |
| Rate per 100 patient-years (95% CI) | 7.5 (6.9-8.2) | 9.9 (9.2-10.7) | 12.4 (11.6-13.2) |
| Unadjusted HR (95% CI)^a^ | 1.00 (Ref.) | 1.38 (1.23-1.55) | 1.77 (1.58-1.98) |
| Additional adjusted HR (95% CI)^b^ | 1.00 (Ref.) | 1.30 (1.16-1.46) | 1.63 (1.44-1.83) |
| **First HF hospitalization** |  |  |  |
| Number of events (%) | 310 (8.7) | 470 (11.8) | 610 (14.7) |
| Rate per 100 patient-years (95% CI) | 4.9 (4.3-5.4) | 6.6 (6.0-7.2) | 8.3 (7.7-9.0) |
| Unadjusted HR (95% CI)^a^ | 1.00 (Ref.) | 1.36 (1.17-1.57) | 1.74 (1.51-2.00) |
| Additional adjusted HR (95% CI)^b^ | 1.00 (Ref.) | 1.27 (1.10-1.47) | 1.58 (1.37-1.83) |
| **CV death** |  |  |  |
| Number of events (%) | 235 (6.6) | 370 (9.3) | 471 (11.3) |
| Rate per 100 patient-years (95% CI) | 3.5 (3.1-4.0) | 4.9 (4.4-5.4) | 5.9 (5.4-6.5) |
| Unadjusted HR (95% CI)^a^ | 1.00 (Ref.) | 1.54 (1.30-1.81) | 1.99 (1.69-2.34) |
| Additional adjusted HR (95% CI)^b^ | 1.00 (Ref.) | 1.47 (1.25-1.74) | 1.86 (1.57-2.20) |
| **Non-CV death** |  |  |  |
| Number of events (%) | 67 (1.9) | 100 (2.5) | 159 (3.8) |
| Rate per 100 patient-years (95% CI) | 1.0 (0.8-1.3) | 1.3 (1.1-1.6) | 2.0 (1.7-2.3) |
| Unadjusted HR (95% CI)^a^ | 1.00 (Ref.) | 1.28 (0.93-1.74) | 1.96 (1.46-2.63) |
| Additional adjusted HR (95% CI)^b^ | 1.00 (Ref.) | 1.25 (0.91-1.71) | 1.91 (1.41-2.60) |
| **All-cause death** |  |  |  |
| Number of events (%) | 320 (8.9) | 494 (12.4) | 670 (16.1) |
| Rate per 100 patient-years (95% CI) | 4.8 (4.3-5.3) | 6.5 (5.9-7.1) | 8.4 (7.8-9.1) |
| Unadjusted HR (95% CI)^a^ | 1.00 (Ref.) | 1.45 (1.26-1.68) | 1.98 (1.72-2.27) |
| Additional adjusted HR (95% CI)^b^ | 1.00 (Ref.) | 1.4 (1.22-1.62) | 1.87 (1.62-2.16) |
| **Total HF hospitalizations/CV death** |  |  |  |
| Number of events | 739 | 1219 | 1645 |
| Rate per 100 patient-years (95% CI) | 11.1 (10.0-12.2) | 16.0 (14.7-17.4) | 20.7 (19.2-22.3) |
| Unadjusted RR (95% CI)^c^ | 1.00 (Ref.) | 1.63 (1.42-1.88) | 2.38 (2.07-2.73) |
| Adjusted RR (95% CI)^b^ | 1.00 (Ref.) | 1.53 (1.33-1.75) | 2.19 (1.90-2.52) |

Definitions and abbreviations as in **Table 3**.

Supplementary Table 19: Effect of randomized treatment on outcomes according to baseline EuroQol 5-Dimension 3-Level (EQ-5D-3L) questionnaire Level Sum Score (LSS) category divided by tertile

|  | **Tertile 1: 5** | | **Tertile 2: 6-7** | | **Tertile 3: 8-15** | |  |
| --- | --- | --- | --- | --- | --- | --- | --- |
|  | **Sacubitril/**  **Valsartan** | **Active RAS comparator^d^** | **Sacubitril/**  **Valsartan** | **Active RAS comparator^d^** | **Sacubitril/**  **Valsartan** | **Active RAS comparator^d^** | **Interaction**  ***P-*Value** |
| N | 1838 | 1835 | 2367 | 2373 | 2280 | 2281 |  |
| **CV death or HF hospitalization** |  |  |  |  |  |  |  |
| N (%) | 340 (18.5) | 385 (21.0) | 494 (20.9) | 595 (25.1) | 580 (25.4) | 656 (28.8) |  |
| Rate per 100 patient-years (95%CI) | 8.0 (7.2-8.9) | 9.3 (8.4-10.2) | 9.0 (8.2-9.8) | 11.0 (10.2-12.0) | 11.4 (10.5-12.3) | 13.2 (12.2-14.2) |  |
| Unadjusted HR (95%CI)^a^ | 0.87 (0.75-1.00) | | 0.80 (0.71-0.90) | | 0.87 (0.77-0.97) | | 0.59 |
| Adjusted HR (95%CI)^b^ | 0.85 (0.74-0.99) | | 0.80 (0.71-0.90) | | 0.88 (0.78-0.98) | | 0.50 |
| **First HF hospitalization** |  |  |  |  |  |  |  |
| N (%) | 212 (11.5) | 246 (13.4) | 348 (14.7) | 392 (16.5) | 366 (16.1) | 422 (18.5) |  |
| Rate per 100 patient-years (95%CI) | 5.0 (4.4-5.7) | 5.9 (5.2-6.7) | 6.3 (5.7-7.0) | 7.3 (6.6-8.0) | 7.2 (6.5-7.9) | 8.5 (7.7-9.3) |  |
| Unadjusted HR (95%CI)^a^ | 0.85 (0.70-1.02) | | 0.85 (0.74-0.99) | | 0.84 (0.73-0.97) | | 0.99 |
| Adjusted HR (95%CI)^b^ | 0.83 (0.69-1.00) | | 0.85 (0.73-0.98) | | 0.86 (0.75-0.99) | | 0.97 |
| **CV death** |  |  |  |  |  |  |  |
| N (%) | 179 (9.7) | 209 (11.4) | 240 (10.1) | 322 (13.6) | 331 (14.5) | 361 (15.8) |  |
| Rate per 100 patient-years (95%CI) | 4.0 (3.4-4.6) | 4.7 (4.1-5.4) | 4.0 (3.6-4.6) | 5.5 (4.9-6.1) | 6.0 (5.3-6.6) | 6.5 (5.9-7.2) |  |
| Unadjusted HR (95%CI)^a^ | 0.85 (0.69-1.04) | | 0.73 (0.61-0.86) | | 0.92 (0.80-1.07) | | 0.11 |
| Adjusted HR (95%CI)^b^ | 0.84 (0.69-1.03) | | 0.74 (0.62-0.87) | | 0.94 (0.81-1.09) | | 0.12 |
| **Non-CV death** |  |  |  |  |  |  |  |
| N (%) | 54 (2.9) | 55 (3.0) | 72 (3.0) | 75 (3.2) | 91 (4.0) | 94 (4.1) |  |
| Rate per 100 patient-years (95%CI) | 1.2 (0.5-1.6) | 1.2 (0.9-1.6) | 1.2 (1.0-1.5) | 1.3 (1.0-1.6) | 1.6 (1.3-2.0) | 1.7 (1.4-2.1) |  |
| Unadjusted HR (95%CI)^a^ | 0.97 (0.66-1.41) | | 0.94 (0.68-1.29) | | 0.96 (0.72-1.29) | | 0.99 |
| Adjusted HR (95%CI)^b^ | 0.95 (0.65-1.39) | | 0.94 (0.68-1.30) | | 0.92 (0.68-1.23) | | 1.00 |
| **All-cause death** |  |  |  |  |  |  |  |
| N (%) | 251 (13.7) | 276 (15.0) | 333 (14.1) | 418 (17.6) | 453 (19.9) | 472 (20.7) |  |
| Rate per 100 patient-years (95%CI) | 5.6 (4.9-6.3) | 6.2 (5.5-7.0) | 5.6 (5.0-6.2) | 7.1 (6.5-7.9) | 8.1 (7.4-8.9) | 8.5 (7.8-9.3) |  |
| Unadjusted HR (95%CI)^a^ | 0.90 (0.76-1.07) | | 0.78 (0.67-0.90) | | 0.97 (0.85-1.10) | | 0.09 |
| Adjusted HR (95%CI)^b^ | 0.89 (0.75-1.06) | | 0.79 (0.68-0.91) | | 0.97 (0.85-1.10) | | 0.11 |
| **Total HF hospitalizations and CV death** |  |  |  |  |  |  |  |
| N | 519 | 602 | 820 | 1009 | 928 | 1103 |  |
| Rate per 100 patient-years (95%CI) | 11.5 (10.1-13.1) | 13.5 (12.0-15.2) | 13.8 (12.4-15.3) | 17.2 (15.7-19.0) | 16.7 (15.1-18.4) | 19.9 (18.1-21.8) |  |
| Unadjusted RR (95%CI)^c^ | 0.85 (0.70-1.03) | | 0.71 (0.61-0.84) | | 0.85 (0.73-0.99) | | 0.25 |
| Adjusted RR (95%CI)^b^ | 0.84 (0.69-1.01) | | 0.72 (0.62-0.84) | | 0.82 (0.71-0.94) | | 0.38 |

1. Baseline model stratified by study and study-specific geographic region.
2. Further adjusted for age, sex, heart rate, SBP, BMI, NYHA functional class III/IV, LVEF, eGFR, NT-proBNP (log-transformed), atrial fibrillation, ischemic etiology, myocardial infarction, and stroke.
3. Adjusted for geographic region and study
4. Enalapril or Valsartan

BMI, body mass index; CI, confidence interval; CV, cardiovascular; eGFR, estimated glomerular filtration rate; HF, heart failure; hosp. hospitalization; HR, hazard ratio; LVEF, left ventricular ejection fraction; NT-proBNP, N-terminal pro B-type natriuretic peptide; NYHA, New York Heart Association; SBP, systolic blood pressure.

Supplementary Figure 1: Distribution of answers to each question of the EuroQol 5-Dimension 3-Level (EQ-5D-3L) health questionnaire.


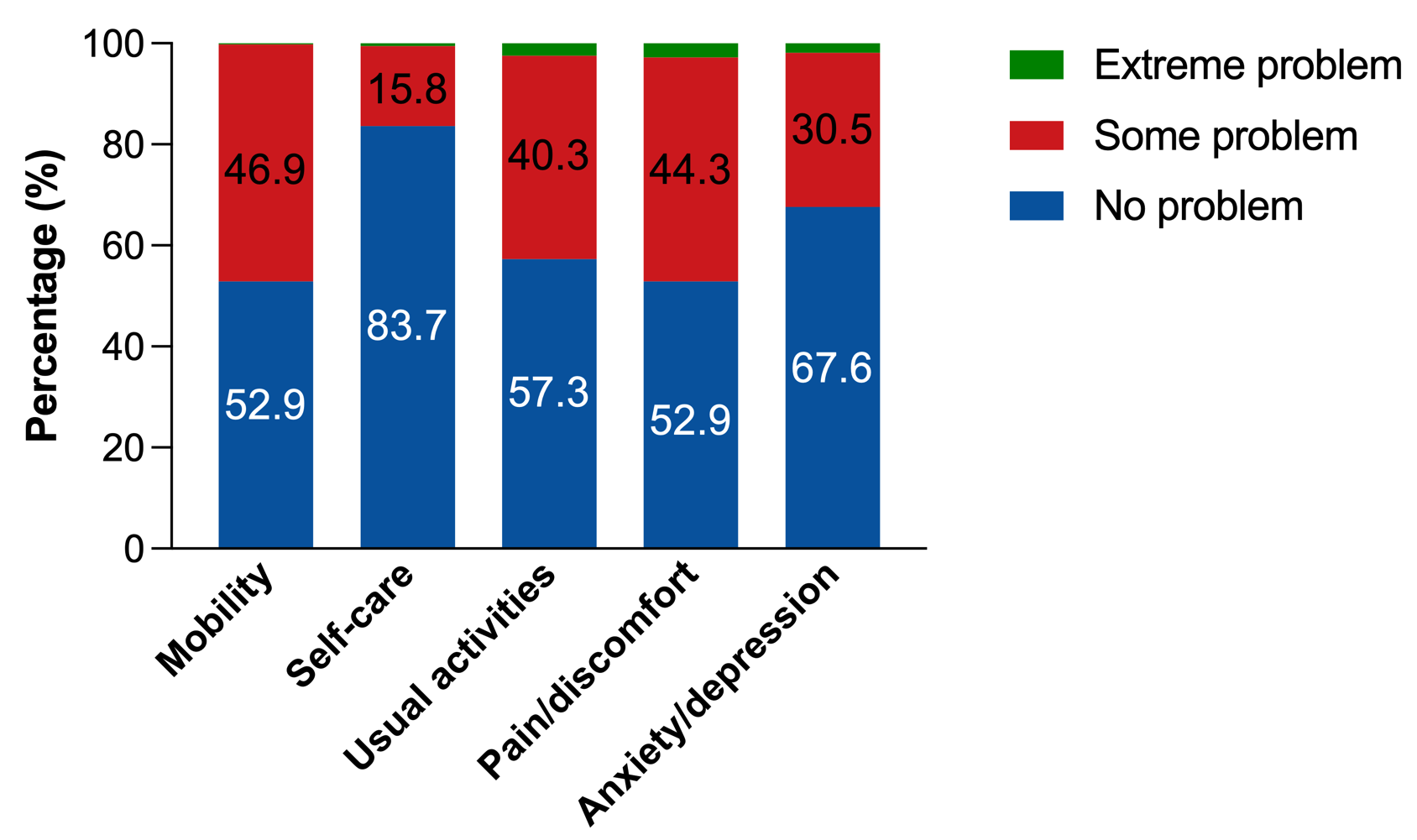


|  | **Mobility** | **Self-care** | **Usual activities** | **Pain/discomfort** | **Anxiety/depression** |
| --- | --- | --- | --- | --- | --- |
| **Extreme**  **problem** | 0.2 | 0.6 | 2.5 | 2.8 | 1.9 |

Data are presented as percentages (%).

Supplementary Figure 2: Distribution of EuroQol 5-Dimension 3-Level (EQ-5D-3L) Level Sum Score (LSS).


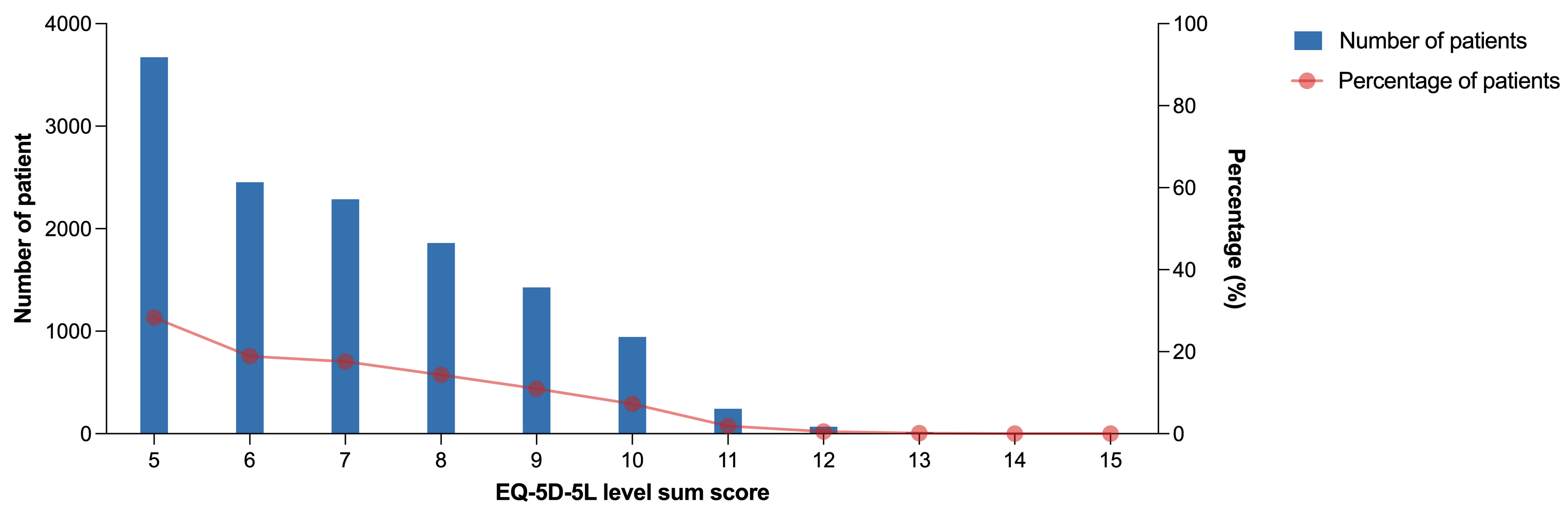


Supplementary Figure 3: Distribution and changes in answers to the EuroQol 5-Dimension 3-Level (EQ-5D-3L) “mobility” question from baseline to 8 months follow-up. (A) Distribution and Sankey plot for the active comparator group; (B) Distribution and Sankey plot for the Sacubitril/Valsartan group. Only values $\geq$50 in the subgroups were shown in the Sankey plot. Bars represent the number of patients in each EQ-5D-3L category. Colors indicate the distribution of EQ-5D-3L scores at randomization and after 8 months of follow-up. Patients with missing EQ-5D-3L assessments are not displayed.


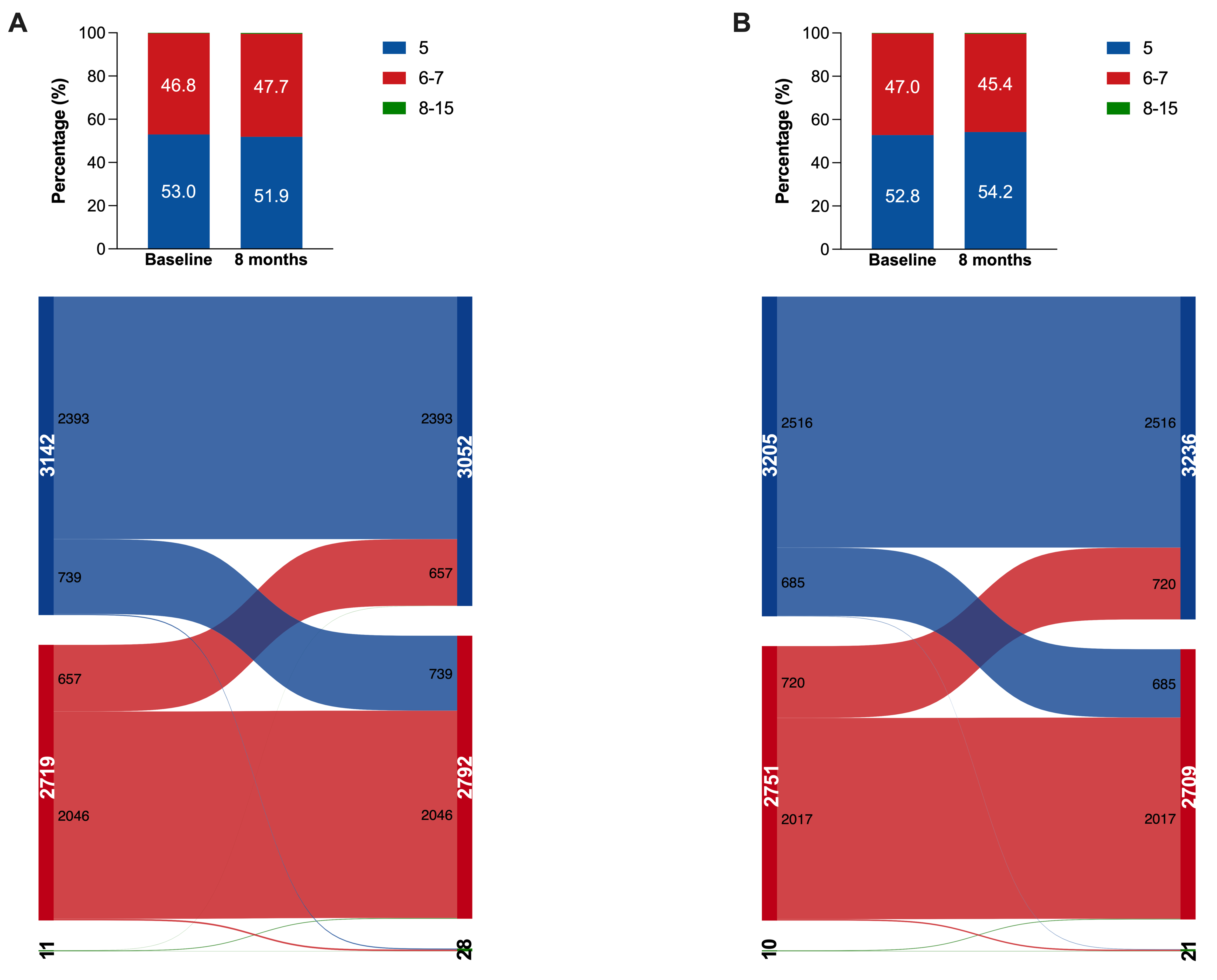


Supplementary Figure 4: Distribution and changes in answers to the EuroQol 5-Dimension 3-Level (EQ-5D-3L) “self-care” question from baseline to 8 months follow-up. (A) Distribution and Sankey plot for the active comparator group; (B) Distribution and Sankey plot for the Sacubitril/Valsartan group. Only values $\geq$50 in the subgroups were shown in the Sankey plot. Bars represent the number of patients in each EQ-5D-3L category. Colors indicate the distribution of EQ-5D-3L scores at randomization and after 8 months of follow-up. Patients with missing EQ-5D-3L assessments are not displayed.


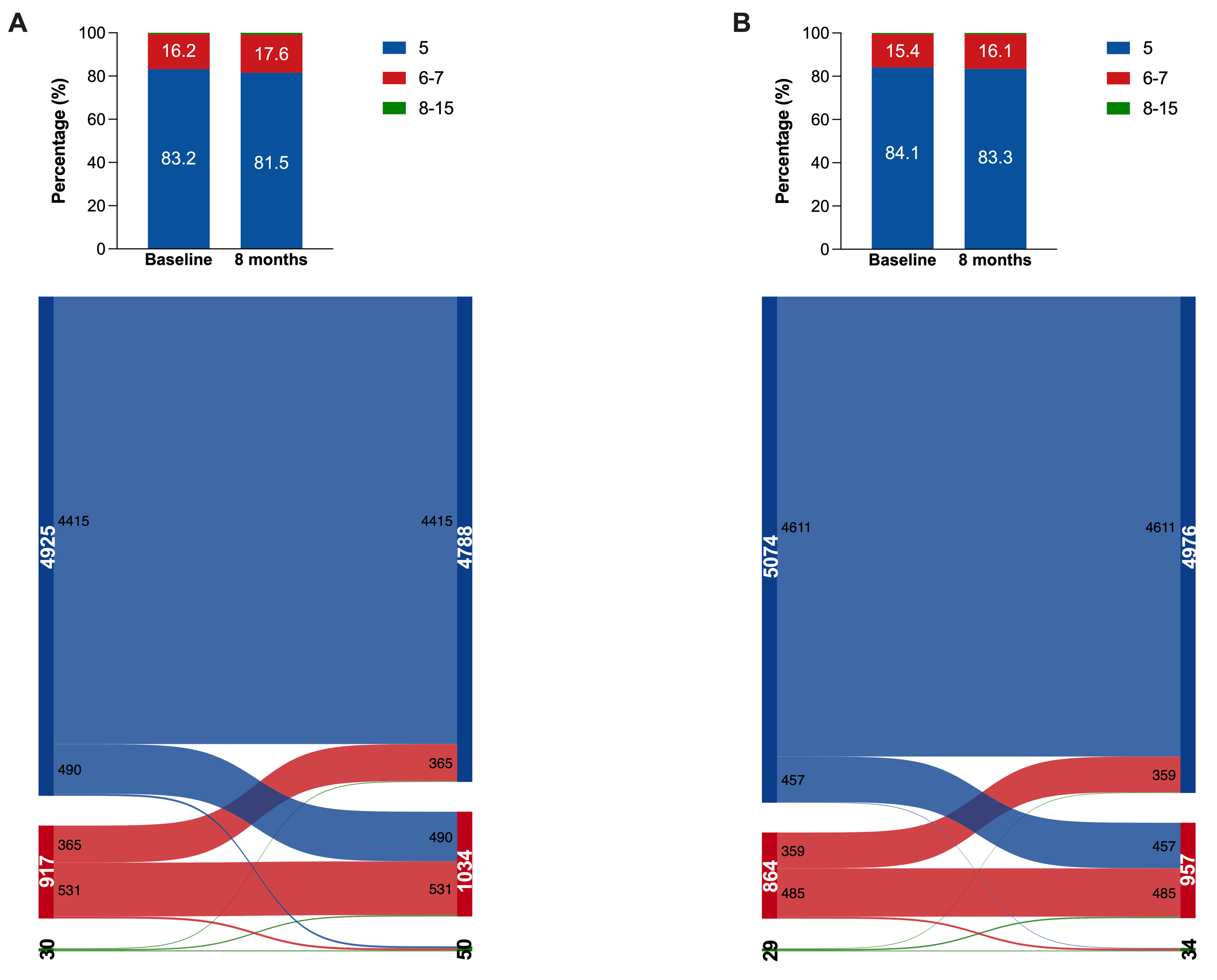


Supplementary Figure 5: Distribution and changes in answers to the EuroQol 5-Dimension 3-Level (EQ-5D-3L) “usual activities” question from baseline to 8 months follow-up. (A) Distribution and Sankey plot for the active comparator group; (B) Distribution and Sankey plot for the Sacubitril/Valsartan group. Only values $\geq$50 in the subgroups were shown in the Sankey plot. Bars represent the number of patients in each EQ-5D-3L category. Colors indicate the distribution of EQ-5D-3L scores at randomization and after 8 months of follow-up. Patients with missing EQ-5D-3L assessments are not displayed.


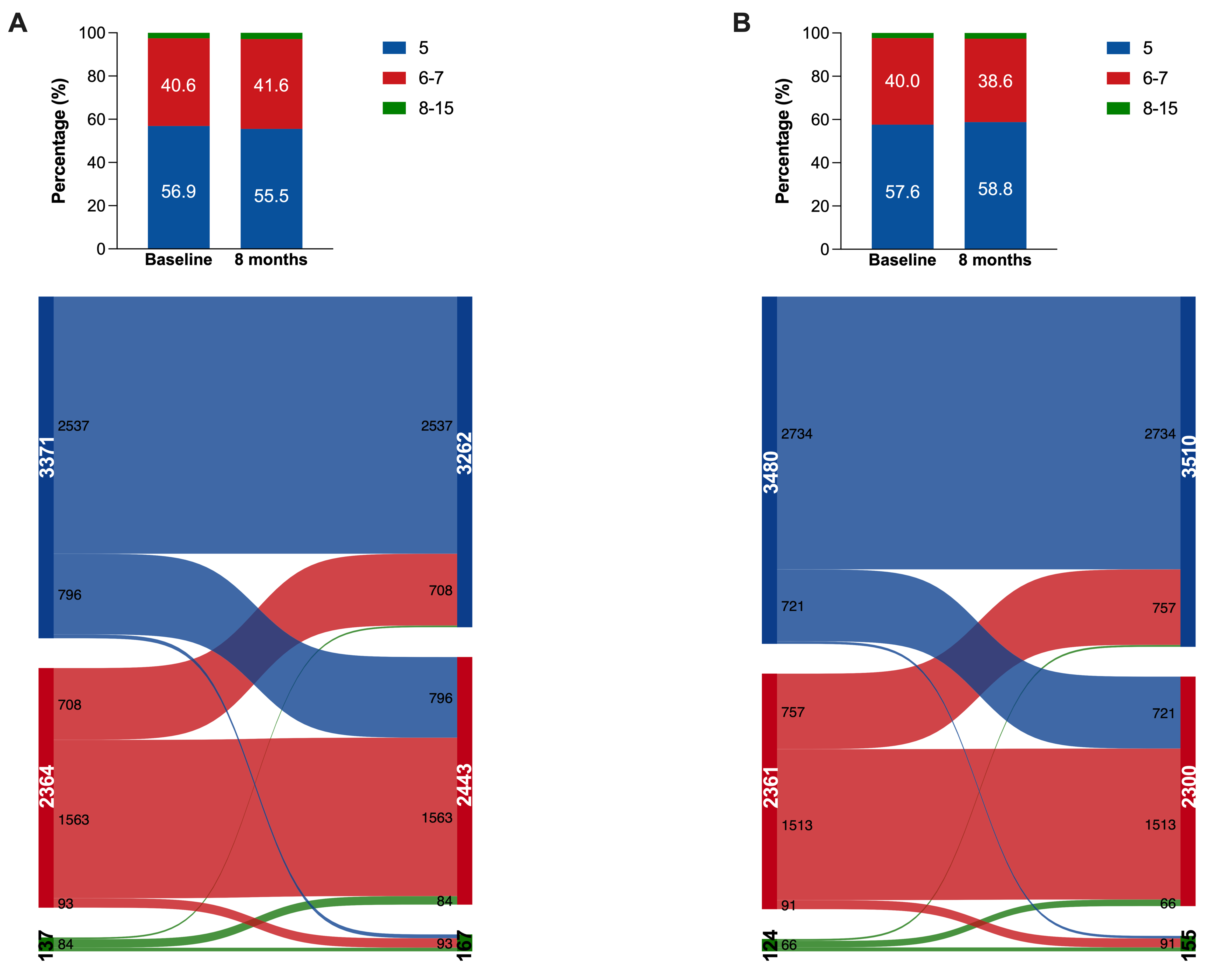


Supplementary Figure 6**:** Effect of sacubitril/valsartan versus active comparator on EuroQol 5-Dimension 3-Level (EQ-5D-3L) questionnaire dimension over time (8 months)


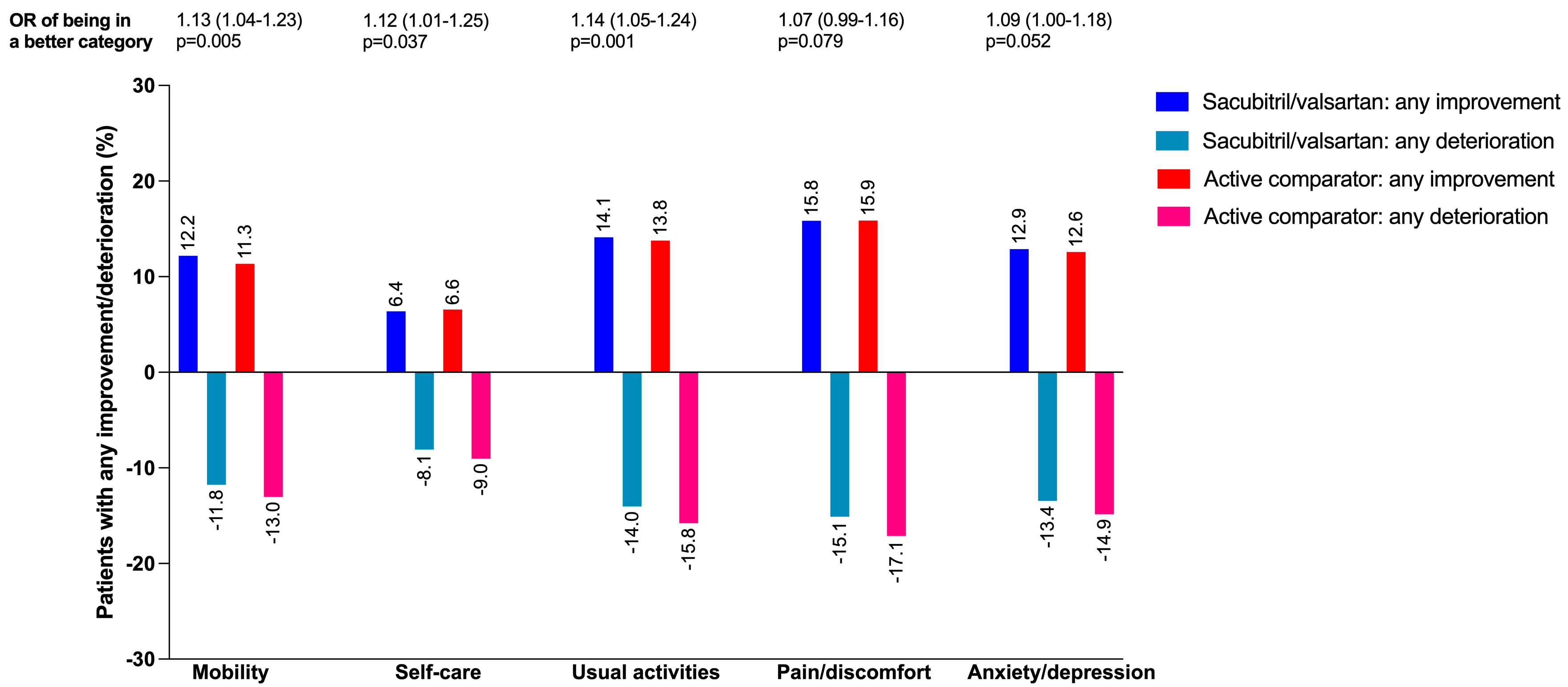


Supplementary Figure 7: Effect of Sacubitril/Valsartan versus active comparator on EuroQol 5-Dimension 3-Level (EQ-5D-3L) Level Sum Score (LSS) over time (8 months) in patients with HF. Values are shown as the percentage of participants experienced any improvement or deterioration in each group. The analysis was adjusted by baseline EQ-5D-3L-LSS.


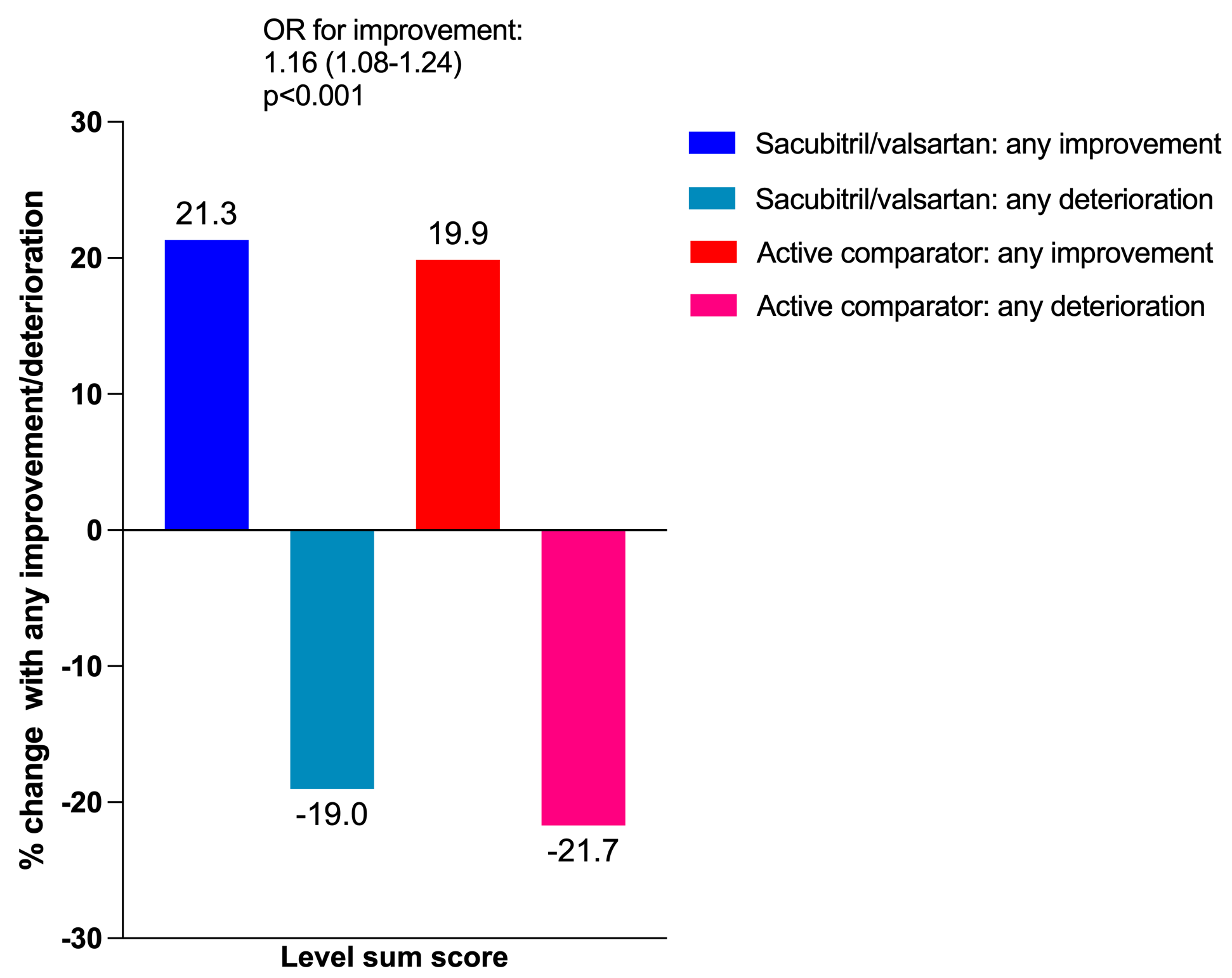


Supplementary Figure 8: Effect of Sacubitril/Valsartan versus active comparator on EuroQol 5-Dimension 3-Level (EQ-5D-3L) Level Sum Score (LSS) over time (8 months) by sex. (A) Male; (B) Female. Values are shown as the percentage of participants experienced any improvement or deterioration in each group. The analysis was adjusted by baseline EQ-5D-3L-LSS.


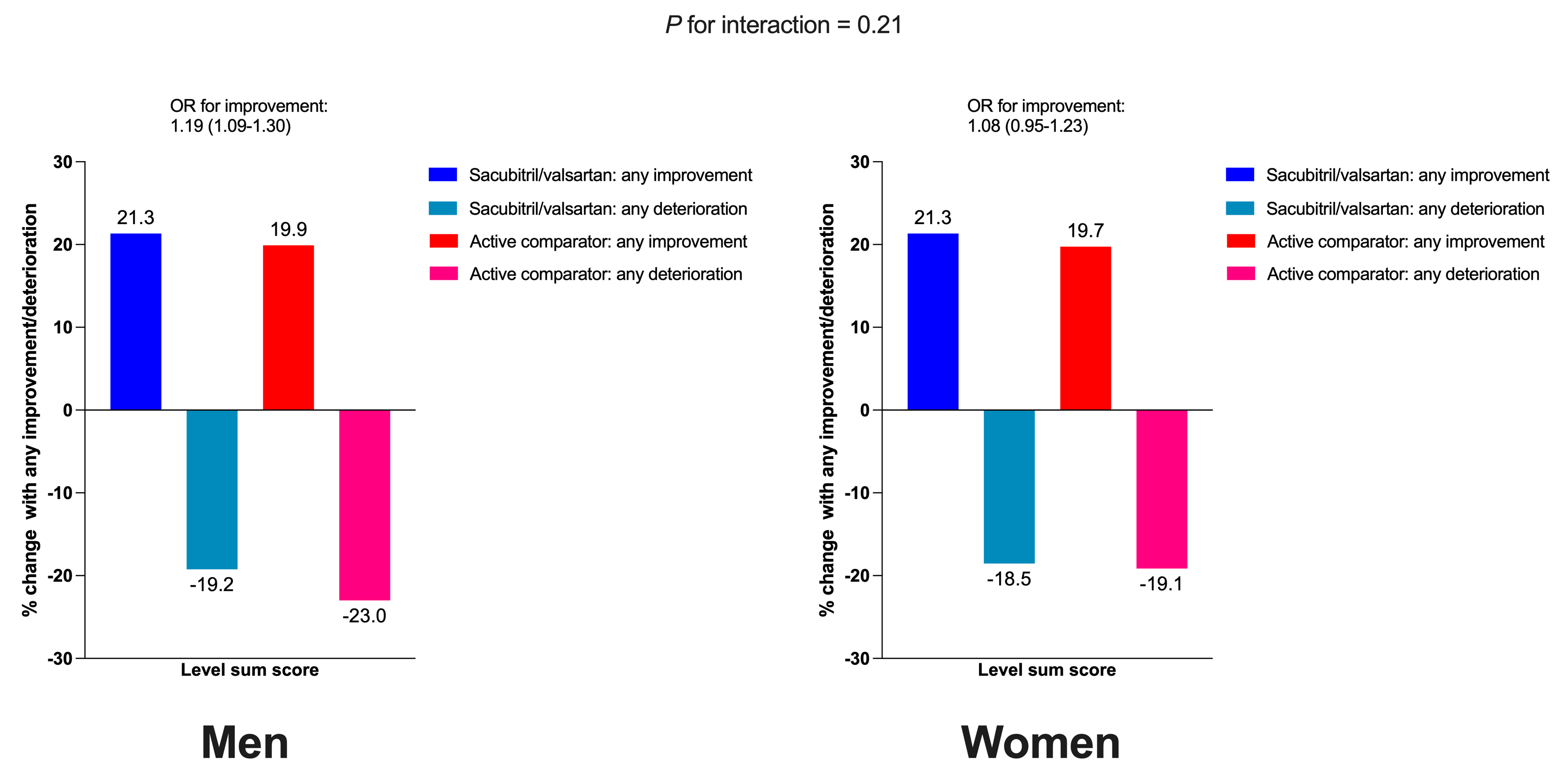


Supplementary Figure 9: Cumulative incidence of first heart failure (HF) hospitalization according to baseline EuroQol 5-Dimension 3-Level (EQ-5D-3L) questionnaire. (A) “Mobility” domain; (B) “self-care” domain; (C) “usual activities” domain; (D) “pain/discomfort” domain; (E) “anxiety/depression” domain; (F) Level Sum Score (LSS) category divided by tertile; (G) Visual Analog Scale (VAS) category divided by tertile. The patients reported “Some problem” and “Extreme problem” were combined into one group due to a small number in the worst category of “mobility” domain (n=27) and “self-care” domain (n=73).


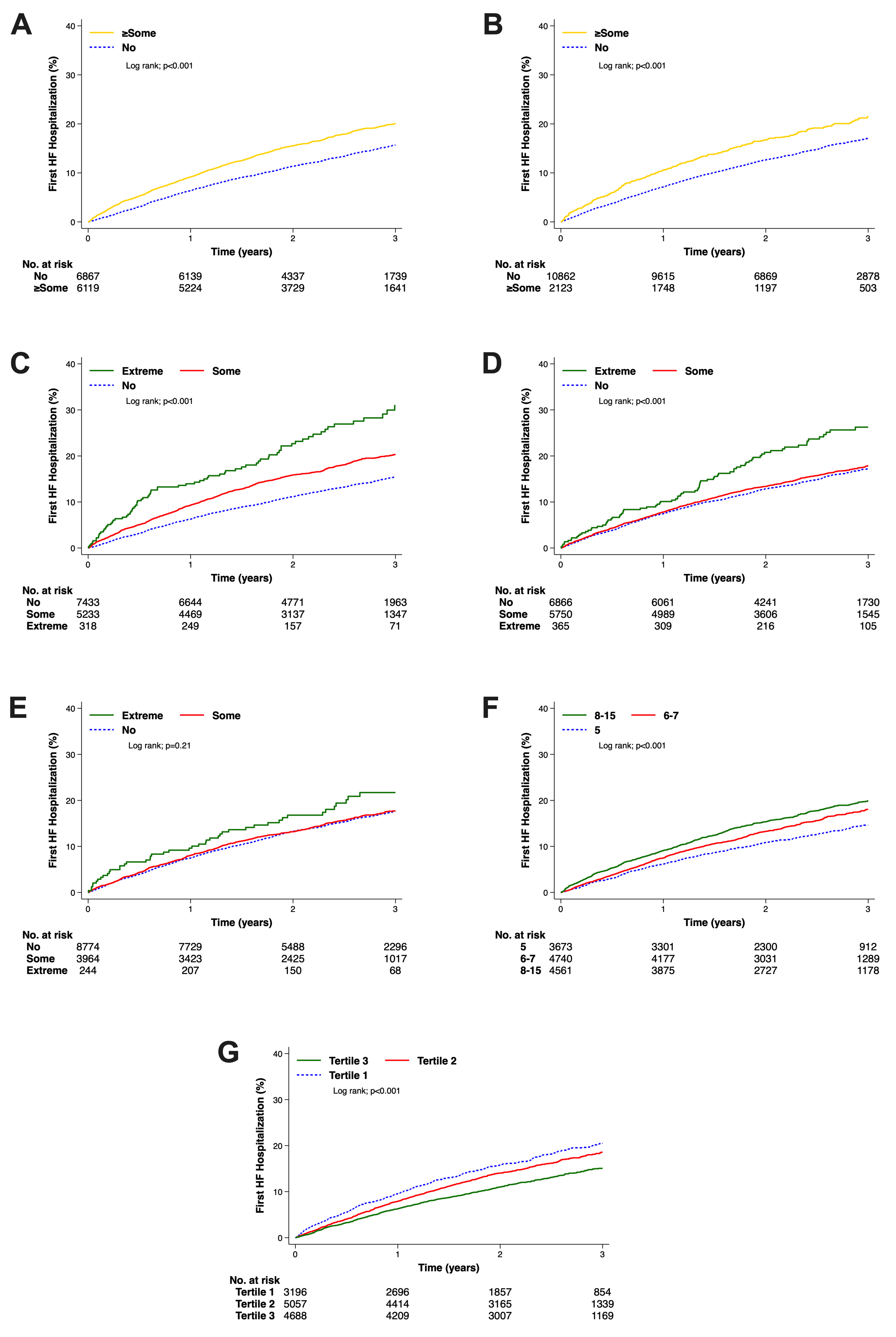


Supplementary Figure 10: Cumulative incidence of cardiovascular (CV) death according to baseline EuroQol 5-Dimension 3-Level (EQ-5D-3L) questionnaire. (A) “Mobility” domain; (B) “self-care” domain; (C) “usual activities” domain; (D) “pain/discomfort” domain; (E) “anxiety/depression” domain; (F) Level Sum Score (LSS) category divided by tertile; (G) Visual Analog Scale (VAS) category divided by tertile. The patients reported “Some problem” and “Extreme problem” were combined into one group due to a small number in the worst category of “mobility” domain (n=27) and “self-care” domain (n=73).


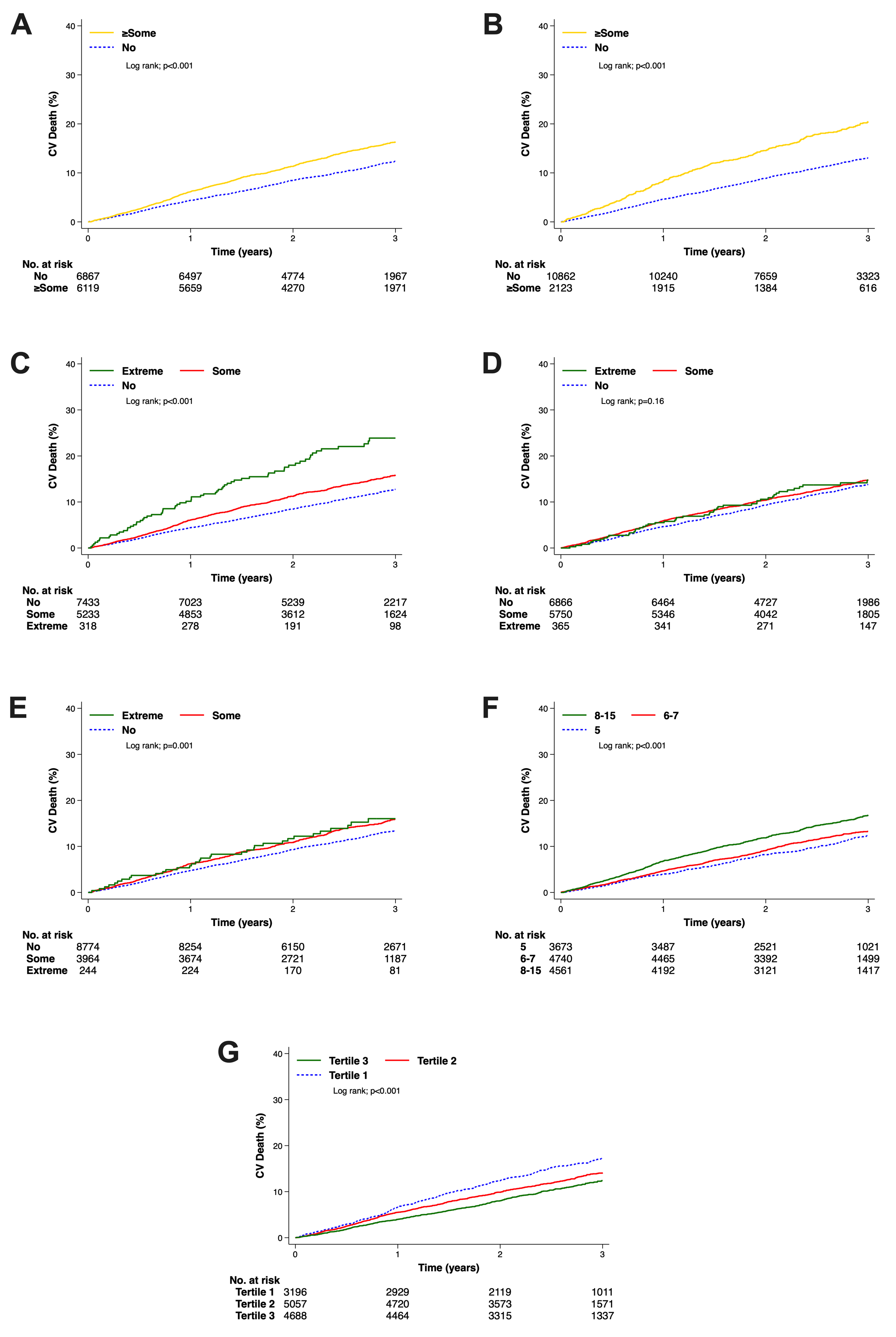

Supplement: pvaf064_Supplementary_Data [file pvaf064_supplementary_data.docx]
